# Identification of multiple integrin β1 homologs in zebrafish (*Danio rerio*)

**DOI:** 10.1186/1471-2121-7-24

**Published:** 2006-06-20

**Authors:** A Paul Mould, Jennifer A McLeish, Julie Huxley-Jones, Alexander C Goonesinghe, Adam FL Hurlstone, Raymond P Boot-Handford, Martin J Humphries

**Affiliations:** 1 Wellcome Trust Centre for Cell-Matrix Research, Faculty of Life Sciences, Michael Smith Building, University of Manchester, Oxford Road, Manchester M13 9PT, UK; 2 Faculty of Life Sciences, Michael Smith Building, University of Manchester, Oxford Road, Manchester M13 9PT, UK

## Abstract

**Background:**

Integrins comprise a large family of α,β heterodimeric, transmembrane cell adhesion receptors that mediate diverse essential biological functions. Higher vertebrates possess a single β1 gene, and the β1 subunit associates with a large number of α subunits to form the major class of extracellular matrix (ECM) receptors. Despite the fact that the zebrafish (*Danio rerio*) is a rapidly emerging model organism of choice for developmental biology and for models of human disease, little is currently known about β1 integrin sequences and functions in this organism.

**Results:**

Using RT-PCR, complete coding sequences of zebrafish β1 paralogs were obtained from zebrafish embryos or adult tissues. The results show that zebrafish possess two β1 paralogs (β1–1 and β1–2) that have a high degree of identity to other vertebrate β1 subunits. In addition, a third, more divergent, β1 paralog is present (β1–3), which may have altered ligand-binding properties. Zebrafish also have other divergent β1-like transcripts, which are C-terminally truncated forms lacking the transmembrane and cytoplasmic domains. Together with β1–3 these truncated forms comprise a novel group of β1 paralogs, all of which have a mutation in the ADMIDAS cation-binding site. Phylogenetic and genomic analyses indicate that the duplication that gave rise to β1–1 and β1–2 occurred after the divergence of the tetrapod and fish lineages, while a subsequent duplication of the ancestor of β1–2 may have given rise to β1–3 and an ancestral truncated paralog. A very recent tandem duplication of the truncated β1 paralogs appears to have taken place. The different zebrafish β1 paralogs have varied patterns of temporal expression during development. β1–1 and β1–2 are ubiquitously expressed in adult tissues, whereas the other β1 paralogs generally show more restricted patterns of expression.

**Conclusion:**

Zebrafish have a large set of integrin β1 paralogs. β1–1 and β1–2 may share the roles of the solitary β1 subunit found in other vertebrates, whereas β1–3 and the truncated β1 paralogs may have acquired novel functions.

## Background

Integrins are a family of metazoan cell surface receptors that play critical roles in cell adhesion, migration, differentiation and survival [1]. Integrins are heterodimeric glycoproteins containing non-covalently associated α and β subunits, and are grouped into sub-families according to the identity of the β subunit. In humans, eight different β subunits combine with 18 different α subunits to form 24 functionally distinct heterodimers. Integrins have a large extracellular domain responsible for interacting with extracellular ligands, and a small intracellular domain that binds to cytoskeletal and signaling proteins. Integrins assimilate information from the extracellular and intracellular environments by acting as bi-directional transducers of signals across the cell membrane. Hence, the binding of an extracellular ligand can elicit activation of intracellular signaling pathways and reorganisation of the cytoskeleton [2,3]. Conversely, changes in intracellular signaling can result in the stimulation or inhibition of ligand binding due to conformational changes in the extracellular domains of an integrin [4-6].

Integrin ligands include the major ECM components laminins, fibronectin and collagens. The β1 integrin sub-family contains 12 different heterodimers in mammals, which form the major group of cell-ECM receptors. An ECM-binding β chain is probably the most ancient, in evolutionary terms, of all the integrin β chains [7,8]. β1 integrins have widespread essential functions both during development and in the adult organism [9-12].

**Figure 1:**
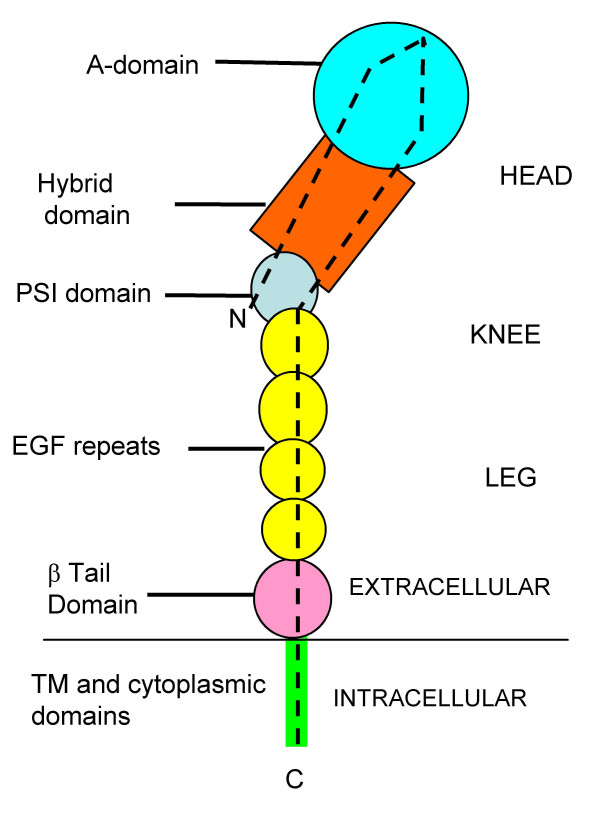
Schematic diagram of the domain structure of integrin β subunits. Domain arrangement is based on crystal structures of the extracellular regions of α Vβ3 [13], α IIbβ3 [14] and β2 [15]. PSI, plexin/semaphorin/integrin; EGF, epidermal growth factor; TM, transmembrane. N-, amino terminus; C-, carboxyl terminus. Dotted line indicates the overall path of the polypeptide chain.

Sequence analysis and X-ray crystal structures [13-15] have demonstrated that all integrin β subunits have an identical domain structure (shown schematically in Fig. 1). At the N-terminus there is a PSI (plexin/semaphorin/integrin) domain, into which is inserted an immunoglobulin fold known as the hybrid domain. A von Willebrand factor type A domain (known as the A or I-like domain) is in turn inserted within the hybrid domain. The PSI domain also links directly to the C-terminal portion of the β subunit, which contains four epidermal growth factor (EGF)-type repeats (EGF-1 to EGF-4), a cystatin-like fold known as the β-terminal domain (βTD), a transmembrane domain and a cytoplasmic tail.

The A domain is the portion of the β subunit that is involved in extracellular ligand binding; this domain also is critical for the interactions with the α subunit that lead to heterodimerisation. The A domain consists of a central β-sheet encircled by seven α helices, and has two large sequence insertions compared to other A domains [16]. These insertions, which are critical for ligand binding and for association with the α subunit, lie in the loops between β-strands B and C and between β-strand D and α helix 5 [13,14]. The βC-βD loop (otherwise known the 'specificity-determining loop') contains a small disulfide-bonded segment that contributes to the differences in ligand-binding specificity between different integrin heterodimers [17-19]. The A domain also contains three cation-binding sites: MIDAS (metal-ion dependent adhesion site), ADMIDAS (adjacent to MIDAS) and LIMBS (ligand-associated metal binding site) [13,14]. The MIDAS site is critical for ligand recognition, whereas the ADMIDAS and LIMBS sites appear to have regulatory roles [20,21].

X-ray crystal structures [13-15] of the extracellular domains have revealed that the overall shape of the β subunit is that of a 'head' connected to a long 'leg' with an intervening 'genu' or 'knee' (Fig. 1). The A domain and hybrid domain form part of the integrin head region, whereas the PSI and EGF-1 and EGF-2 lie in the knee region; the remaining EGF repeats and βTD domain contribute to the leg region. The conversion of an integrin from an inactive to an active state is proposed to involve an unbending of the knee region, and a key hinge point in the knee region has recently been proposed to lie in EGF-2 [15]. A critical feature of this unbending is the release of the hybrid domain from interactions with other integrin domains, thereby allowing the hybrid to undergo an outward pivoting. In turn, this movement causes conformational changes in the A domain that promote ligand recognition [14,22]. The β subunit cytoplasmic tail contains a HDRRE motif for interaction with the α subunit cytoplasmic domain [23], and NPXY sequences that are involved in binding to cytoskeletal proteins such as talin and filamin [24].

The zebrafish is an emerging model organism with a large number of attractive features for studying the function of genes, such as: (i) the external and rapid development of embryos, (ii) the transparency of embryos, which allows visualisation and tracking of individual cells or groups of cells, (iii) the high degree of conservation between zebrafish and human genes, (iv) the similarities between human and zebrafish embryonic development resulting in a large proportion of tissues and organs being grossly similar between the two species, (v) its genetic tractability, aided by the availability of powerful genetic tools such as gene knock-down by morpholino-modified antisense oligonucleotides (morpholinos) [25] and gene knockout by target-selected mutational inactivation of genes (TILLING)[26], and (vi) the partially complete genome sequence [59]. In addition, zebrafish is finding increasing use as a model organism to study human diseases [27,28]; in many of these disorders integrin-ligand interactions play important roles in the initiation or progression of the disease [29].

To date, our knowledge of the biological roles of integrins in vertebrates has been derived mainly from knockout studies in mice [30,31]. For the reasons described above, zebrafish provide a very attractive alternative model system for studying integrin function during development and disease. Currently, however, very little published information is available concerning the sequences and complement of integrin genes in this organism. Here we have investigated if β1-like genes and transcripts are found in zebrafish. We found that zebrafish have two β1 paralogs that are closely related to the single gene found in other vertebrates. Surprisingly, we also found multiple, more divergent, zebrafish β1 paralogs, many of which are truncated and entirely novel forms.

## Results

### Zebrafish have two β1 paralogs (β1–1 and β1–2) that are closely related to human β1

**Figure 2:**
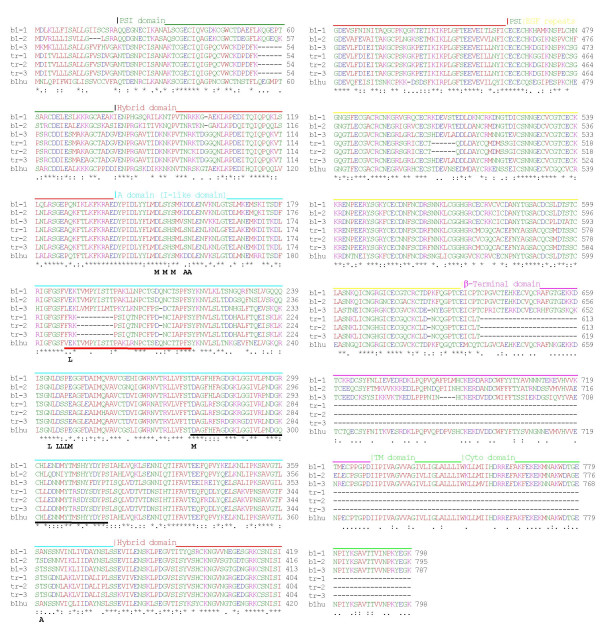
Alignment of zebrafish β1 sequences (β1–1, β1–2, β1–3, β1tr-1, β1tr-2 and β1tr-3) with human β1. The alignment was performed using ClustalW. Protein domains are annotated: PSI, plexin/semaphorin/integrin; EGF, epidermal growth factor; βTD, β terminal domain; TM transmembrane; cyto, cytoplasmic domain. MIDAS (M), ADMIDAS (A) and LIMBS (L) cation binding residues in the A domain are shown. Red underline indicates the position of the βB-βC loop, black underline indicates the position of the βD-α5 loop in the A domain. Sequence identities are indicated by *, conservative substitutions by :, semi-conservative substitutions by.

**Figure 3:**
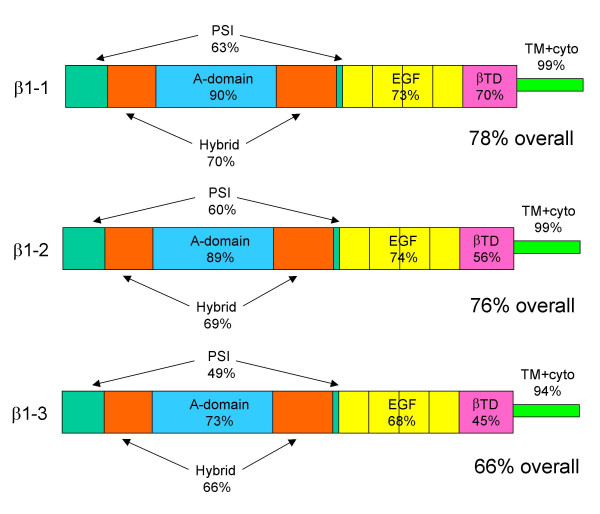
Domain comparison of zebrafish β1 paralogs β1–1 and β1–2 with human β1 (% sequence identity). PSI, plexin/semaphorin/integrin; EGF, epidermal growth factor; βTD, β terminal domain; TM+ cyto, transmembrane and cytoplasmic domains.

To amplify β1-like sequences, primers were designed based on partial sequences in the zebrafish genome project or from EST databases (see Methods). Complete coding sequences were amplified by RT-PCR using RNA from 4-day old embryos. Two of the sequences obtained, designated β1–1 and β1–2, had a high degree of identity to other vertebrate β1 sequences, such as human β1 [Genbank Q8WUM6] (Fig. 2). These two sequences are >76% identical to human β1 overall but two regions of the subunits are extremely well conserved (Fig. 3A,B). The first of these regions is the A domain, indicating that the key functional features of this domain are retained in these β subunits. Highly conserved portions of the A domain include the loops important for ligand binding and association with the α subunits (Fig. 2). All residues involved in the coordination of the MIDAS, ADMIDAS and LIMBS cations are also completely conserved (Fig. 2). The second region of very strong conservation is the transmembrane domain and cytoplasmic tail, suggesting that the signaling and intracellular ligand binding properties of these sequences have been highly conserved. The conserved features include the HDRRE α subunit cytoplasmic domain interaction sequence and the two NPXY motifs [23,24]. All 56 cysteine residues in the extracellular domains are also perfectly conserved between β1–1, β1–2 and human β1. The sequences of β1–1 and β1–2 are 81% identical to each other; β1–2 is 84% identical to a published catfish β1 sequence [32] [Genbank:Q9AI01].

### Zebrafish have a third β1 paralog (β1–3), which is more divergent in sequence from human β1

A third sequence amplified from zebrafish embryos, designated β1–3, was considerably more divergent in sequence from human β1 than β1–1 and β1–2 (Fig. 3 and Table 1). β1–3 also has fewer potential N-linked glycosylation sites (five) than β1–1 and β1–2 (which both have eight). However, as observed for β1–1 and β1–2, all 56 cysteine residues are perfectly conserved. Overall, β1–3 is more closely related in sequence to β1–2 than to β1–1 (Table 1). In comparison to the very high degree of sequence conservation seen for the A domains of β1–1 and β1–2, the A domain of β1–3 is not well conserved (Fig. 3). Although the MIDAS and LIMBS cation-binding sites are conserved in the β1–3 A domain, two aspartate residues are substituted by Ser/Asn at the ADMIDAS site; these two aspartate residues are present in all other chordate β1 subunits. Because each aspartate residue provides two carboxylate oxygen atoms for coordination of the AMIDAS cation, the double substitution will probably lead to loss of cation binding at the ADMIDAS. We have previously shown that mutation of these residues in human β1 causes constitutive inactivation of α5β1 [20]. In addition, the sequence of the 'specificity-determining loop' between β strands B and C differs at several positions to that of the other β1 subunits; most notably, the sequence of the disulfide-linked loop [17] in β1–3 (CFPSDC) is markedly different to that of β1–1 and β1–2, which are both very similar to human β1 sequence (CTSEQNC). Hence, β1–3 is likely to have altered ligand-binding and α subunit association properties compared with β1–1 and β1–2 [17,19]. However, the transmembrane domain and cytoplasmic tail of β1–3 is highly conserved, and this region is therefore likely to retain all the signaling characteristics and intracellular ligand binding features of β1–1 and β1–2.

**Table 1:** Percentage amino acid identities between zebrafish and human β1 sequences

% id	β1–1	β1–2	β1–3	β1tr-1	β1tr-2	β1tr-3	ESTa	ESTb	human
β1–1	-	81	69	62	62	62	62	62	78
β1–2	81	-	74	65	65	66	65	65	76
β1–3	69	74	-	79	79	82	81	80	66
β1tr-1	62	65	79	-	98	95	94	98	59
β1tr-2	62	65	79	98	-	95	94	98	59
β1tr-3	62	66	82	95	95	-	95	95	59
ESTa	62	65	81	94	94	95	-	95	59
ESTb	62	65	80	98	98	95	95	-	59

### Zebrafish have truncated β1 paralogs that are closely related to β1–3

**Figure 4:**
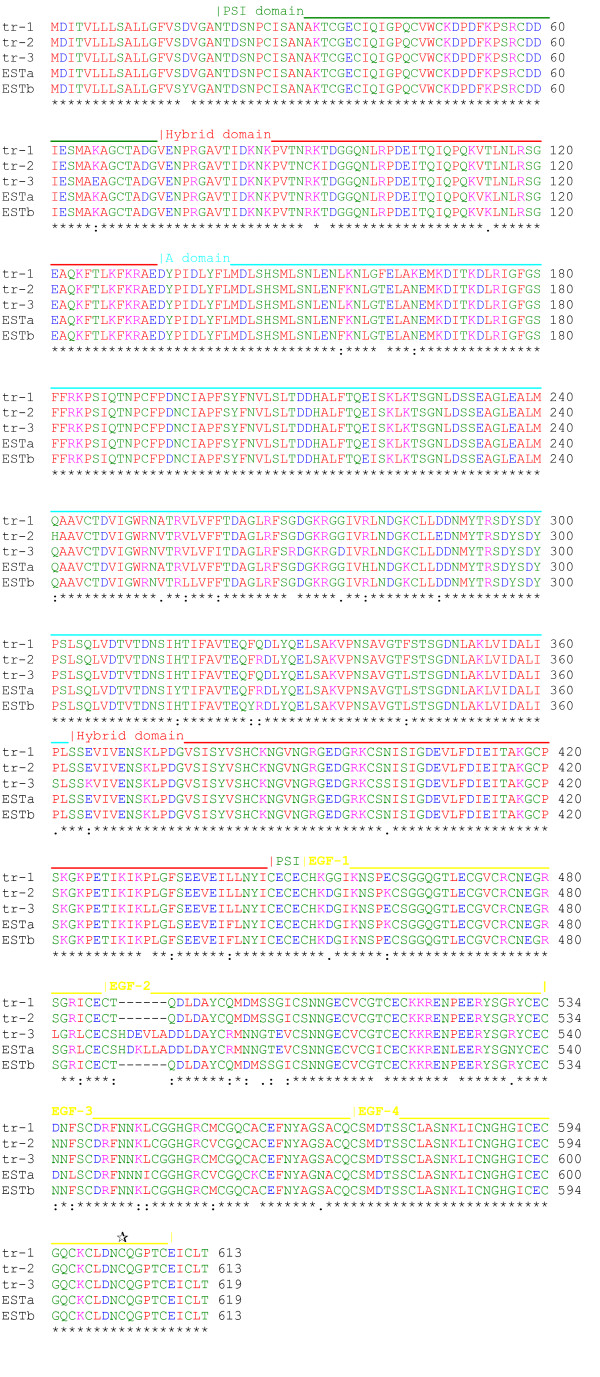
Alignment of zebrafish truncated β1 sequences. The alignment was performed using ClustalW. Protein domains are annotated: PSI, plexin/semaphorin/integrin; EGF, epidermal growth factor. Yellow bar indicates the position of a putative flexible linker region in EGF-2 [15]. Domain boundaries in the EGF repeats are as predicted by Takagi *et al*. [58]. Based on this prediction, the truncated β1 sequences contain four additional residues after the end of EGF-4. The position of an additional cysteine residue in EGF-4 (not seen in other β subunits) is indicated by *tial*. Sequence identities are indicated by *, conservative substitutions by :, semi-conservative substitutions by.

**Figure 5:**
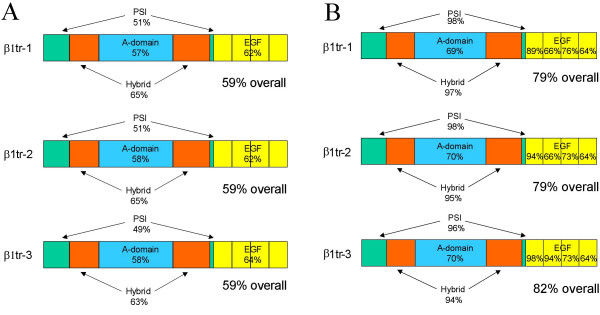
A. Domain comparison of zebrafish truncated β1 paralogs with human β1 (% sequence identity). B. Domain comparison of truncated β1 paralogs with β1–3 (% sequence identity). PSI, plexin/semaphorin/integrin; EGF, epidermal growth factor; βTD, β terminal domain; TM+ cyto, transmembrane and cytoplasmic domains. The sequence identities of the individual EGF repeats are shown in B.

Several zebrafish database partial EST sequences [60] (Table 2) appeared to be orthologous to human β1 but to differ in sequence with β1–1, β1–2 and β1–3. Two of these ESTs, ESTa and ESTb, were selected for complete sequencing. Although the ESTs were found to be full-length cDNA clones extending from the start codon to the polyA tail, the integrin sequence carried a stop codon at the end of the EGF repeats (see Additional file 1). Hence, these transcripts were lacking the βTD, transmembrane and cytoplasmic domains. The coding sequences of the two ESTs were 95% identical to each other at the amino acid level. The two ESTs had almost identical 5' sequences but were more divergent in the 3' region, including the 3'UTR. In order to examine whether similar sequences could be amplified from zebrafish embryos, we performed RT-PCR using a common forward (5') primer but with different reverse (3') primers, which were specific for either ESTa or ESTb (see Additional file 1). PCR products were subcloned and individual clones were sequenced. The most abundant sequence obtained using the ESTa reverse primer from 4-day-old embryo RNA was designated β1tr-1 (truncated β1 paralog 1); the predominant sequence obtained using the ESTb reverse primer was designated β1tr-2 (truncated β1 paralog 2). Since ESTa was obtained from an adult kidney cDNA library, we also used the same primer sets to amplify products from reverse transcribed kidney RNA. The most abundant sequence obtained using the ESTa reverse primer was identical to that of β1tr-1; however a distinct sequence, designated β1tr-3, was the predominant sequence obtained using the ESTb reverse primer. All three of these paralogs carried a stop codon at the same position as that found in the two ESTs (see Additional file 1), resulting in a truncated translation product terminating four residues after the end of EGF-4. Hence, these data clearly establish that this group of paralogs are *bona fide *truncated β1-like subunits. The sequence of the truncated paralogs is shown in Fig. 2, and in alignment with the EST sequences in Fig. 4. The truncated β1 paralogs are 95–98% identical to the EST sequences or to each other (Table 1); β1tr-1 and β1tr-2 are most like ESTb, whereas β1tr-3 has a similar degree of identity to both ESTa and ESTb. The sequence differences between the truncated paralogs were not due to PCR or sequencing errors because the sequences were confirmed in multiple independent PCR products. The truncated paralogs are even more divergent from human β1 than is β1–3 (Table 1, Fig. 5A), and like β1–3, these paralogs have relatively few potential glycosylation sites (β1tr-1 and β1tr-2 both have three sites, β1tr-3 has four). However, all cysteine residues remain perfectly conserved, although an additional cysteine residue is present in EGF-4. This extra cysteine residue could form a disulfide bond with the cysteine residue present in the additional four residues at the C-terminus (Fig. 4).

**Table 2:** Corresponding database entries for zebrafish β1 sequences

Paralog	Accession Number	Gene Prediction	Genomic Location	Corresponding (or additional) EST	Source of EST
β1–1	DQ149101	GENSCAN00000041635	Chr24	DT075719	whole adult
β1–2	DQ149102	GENSCAN00000013230^3^	Chr2^4^	CF417014	gastrula stage
β1–3	DQ149103	GENSCAN00000013230^3^	Chr2^4^	CK711153	<72 h embryos
β1tr-1	DQ440587	-	-	-	-
β1tr-2	DQ440588	-	-	-	-
β1tr-3	DQ440589	-	-	CN328139^5^	liver
ESTa	BC067552^1^	-	-	CN329042^6^	liver
ESTb	DQ440590^2^	GENSCAN00000013230^3^	Chr2^4^	DN836198	pooled gut

^1 ^The complete sequence of ESTa was found to be 100% identical to that of BC067552. A partial sequence of this clone can be found under Genbank Accession number CD285257.

^2 ^A partial sequence of this clone can be found under Genbank Accession number CF347594.

^3 ^The sequences of β1–2, β1–3 and ESTb are all contained within a single (inaccurate) gene prediction 2609 residues in length (genome project Zv5).

^4 ^Latest version of genome project (Zv6).

^5 ^Identical sequence in coding region but 3'UTR sequence appears to differ. Additional EST BG729583 (kidney).

^6 ^Other ESTs: CO916860 (olfactory epithelium), CK865233 (testis), CN315944 (liver), CN171841 (kidney), CN320549 (liver).

All the truncated paralogs are closely related to β1–3 (Fig. 5B), with ~80% sequence identity overall. Hence, together with β1–3 they form a novel group of β1 paralogs. In a surprising contrast to what is observed regarding sequence conservation between β1–1 β1–2 and human β1(Fig. 3), the PSI and hybrid domains are almost perfectly conserved between the truncated paralogs and β1–3, whereas the A domains are much less well conserved (Fig. 5B). In addition, the sequence of EGF-1 is highly conserved, and the first two EGF repeats of β1tr-3 are almost identical in sequence to those of β1–3 (Fig. 5B). The major region of sequence variation between the truncated β1 paralogs is the putative linker sequence in EGF repeat 2 [15], with some of the paralogs having a six amino acid deletion in this region (Fig. 4).

The truncated paralogs, like β1–3, have a substitution of two key aspartate residues by Ser/Asn at the ADMIDAS. In addition, these paralogs are also lacking an essential LIMBS residue (E189 in human β1 [E169 in the mature sequence]) due to a glutamate to arginine substitution. A similar mutation in human β1 (APM and J. Askari, unpublished data) or β3 [33] perturbs ligand recognition. Hence, the A domains of the truncated paralogs may not be functional for ligand binding, or may possibly bind to ligands in a different manner to other β subunit A domains. The MIDAS coordinating residues are conserved in the truncated paralogs; hence, it remains possible that they may bind ligands in a manner akin to the A domains found in some α subunits [6].

Overall, the sequences of the A domains in the truncated paralogs are highly divergent from other chordate sequences; indeed, they are only slightly more similar to human β1 A domain (57–58% identity) than the Ciona β1 A domain (56% identity). The loops important for ligand binding and association with the α subunit are poorly conserved. For example, the truncated paralogs all have a large deletion of nine amino acids in the β B-βC loop. A second part of the α/β subunit interface involves residues in the βD-α5 loop, especially those that form a short stretch of 3_10 _helix (DGKL) [13]. This region is highly conserved between the full-length paralogs and human β1 (Fig. 2) but has several substitutions in the truncated paralogs. Taken together, these findings suggest that the truncated β1 paralogs may have weaker associations with α subunits, associate with different α subunits, or even may not associate with α subunits at all. However, even if the truncated paralogs are able to associate with α subunits, they would form non-signaling receptors, as they lack the transmembrane and cytoplasmic domains.

### Genomic and phylogenetic analysis reveals evolutionary relationships between the β1 paralogs

Gene sequences corresponding to β1–1, β1–2 and β1–3 can be identified in the zebrafish genome (Table 2) (although there are a number of inaccuracies and gaps in the GENESCAN *ab initio *gene predictions). Currently, the gene for only one truncated paralog can be identified from the latest genome sequence (Zv6), corresponding almost exactly to ESTb (Table 2). β1–2, 'ESTb' and β1–3 sequences lie adjacent to one another in a tandem array on chromosome 2; β1–1 lies in a separate region of the genome (chromosome 24). The genomic sequences show almost perfect conservation of exon- intron boundaries (data not shown), suggesting that all the β1 paralogs arose from duplication of a single ancestral β1 gene.

**Figure 6:**
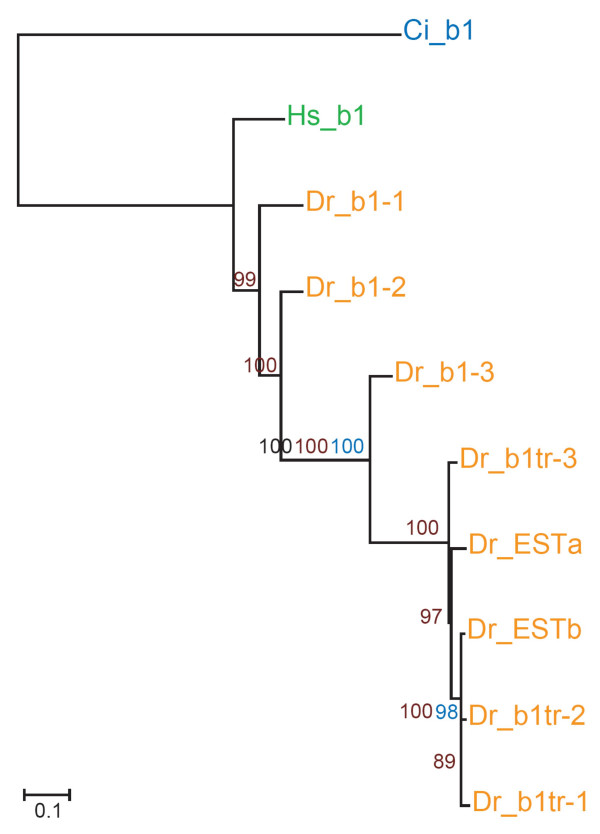
Phylogenetic relationship of zebrafish (Dr) β1 integrin chains with representative deuterostome orthologs. Maximum Likelihood tree is shown with supporting Neighbor Joining bootstrap replicates (black), Maximum Parsimony bootstrap replicates (blue) and Bayesian clade credibility values (brown). Horizontal scale is amino acid replacements per site. *Ciona intestinalis *β1 (Ci_b1) was used to root the tree; *homo sapiens *β1 (Hs_b1) is shown as an example of tetrapod sequence.

For phylogenetic analysis, the six zebrafish β1 sequences and two EST sequences were aligned with human β1 (see Fig. 2 and Additional file 2). The Ciona β1 sequence is ancestral to β1/β2/β7 [8] and is used as an outgroup for the phylogenetic analysis. The resulting phylogenetic analysis is presented in Fig. 6 in the form of a maximum likelihood (ML) tree with supporting data from 1,000 neighbor-joining bootstrap replicates, 1,000 maximum parsimony bootstrap replicates and Bayesian clade credibility values. The phylogeny suggests that: (i) the zebrafish β1 paralogs are all derived from a single ancestral β1 gene present in the last common ancestor of zebrafish and tetrapods, (ii) the duplication that gave rise to β1–1 and β1–2 occurred after the divergence of the tetrapod and teleost lineages, (iii) a subsequent duplication gave rise to the ancestor of the divergent paralogs, (iv) this ancestral divergent paralog then duplicated, giving rise to β1–3 and the ancestor of the truncated paralogs, (v) a very recent tandem duplication of the ancestral truncated paralog has taken place. The branching order of the truncated paralogs has low statistical support, indicative of the high sequence similarity. However, the statistical analysis suggests that ESTb, β1tr-1 and β1tr-2 are more closely related to each other than to ESTa and β1tr-3, and probably arose more recently.

The ML tree was supported by maximum parsimony and Bayesian analyses. Only neighbor joining analysis (not shown) produced two vertebrate clades where β1–1, β1–2 and human β1 grouped separately to the other sequences. However, the genomic locations of the different paralogs add support to the ML analysis. For example, the position of β1–2 and β1–3 in close proximity on the same scaffold suggests that β1–2 and β1–3 arose from a tandem duplication of a common ancestor. The presence of a truncated paralog in the same region of the genome adds weight to the proposal (supported by all the phylogenetic analyses) that the ancestor of the truncated paralogs arose from a duplication of the precursor of β1–3.

### The divergent β1 paralogs are expressed later in development than β1–1 and β1–2

**Figure 7:**
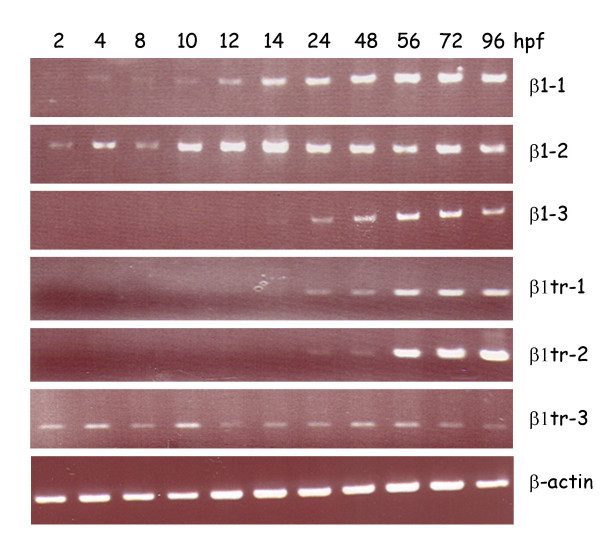
Expression profiling of zebrafish β1 paralogs during development. Expression of the different paralogs at 2, 4, 6, 8, 12, 14, 24, 48, 56, 72 and 96 hpf (hours post fertilization) was analyzed by semiquantitative RT-PCR using primers designed against a portion of extracellular domains of the different paralogs. The amplification of zebrafish β-actin was monitored as a positive control.

We examined the expression of the different β1 paralogs at different times of development using RT-PCR with primers specific for each paralog (Fig. 7). The results showed that both β1–1 and β1–2 were expressed throughout development. The expression of β1–2 appeared to peak at around 14 hours post fertilization (hpf) and declined thereafter. In contrast, the expression of β1–1 appeared to be highest at later stages of development (48 hpf onwards). The more divergent β1 paralog β1–3 was not found to be expressed during early development; β1–3 message was first detected at 14 hpf and expression strongly increased at later stages of development. The truncated paralogs β1tr-1 and β1tr-2 were also expressed only at later stages of development, with message first detected at 24 hpf. In contrast, β1tr-3 was expressed throughout development.

### The divergent β1 paralogs are expressed less widely than β1–1 and β1–2

**Figure 8:**
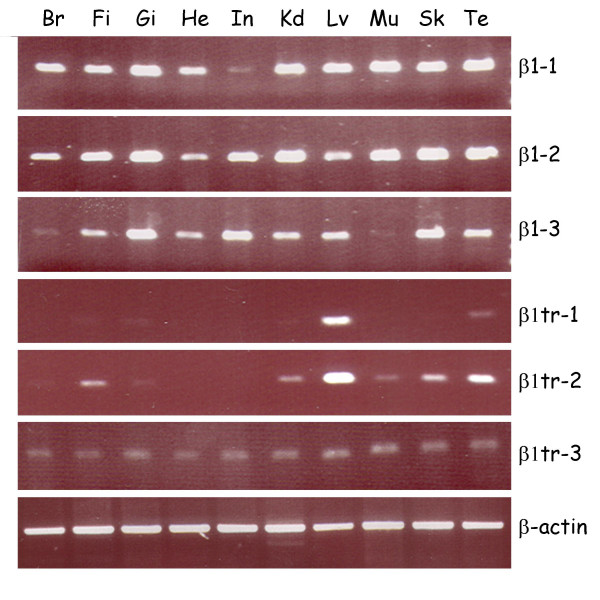
Expression profiling of zebrafish β1 paralogs in adult tissues. Expression of the different paralogs was analyzed by semiquantitative RT-PCR using primers designed against a portion of extracellular domains of the different paralogs. The amplification of zebrafish β-actin was monitored as a positive control. Br, brain; Fi, fin; Gi, gill; He, heart; In, intestine; Kd, kidney; Lv, liver; Mu, muscle; Sk, skin; Te, testis.

The expression of the different β1 paralogs in adult tissues was also examined by RT-PCR (Fig. 8). The results showed that β1–1 and β1–2 were expressed in all tissues, although the expression of β1–1 in intestine was low, whereas β1–2 was highly expressed in this tissue. Expression of β1–3 was widespread, but tissues such as muscle and brain showed only low expression. The truncated paralogs β1tr-1 and β1tr-2 were only expressed in a limited number of tissues, with expression being particularly strong in liver. In contrast to the tissue-restricted expression of β1tr-1 and β1tr-2, β1tr-3 was found to be expressed in all tissues.

EST sequences corresponding to the different β1 paralogs can be identified in the NCBI databases (Table 2). The majority of these EST sequences correspond to those of truncated paralogs. Although no EST sequences that exactly matched β1tr-1 or β1tr-2 were found, ESTs corresponding to the 5' and 3' ends of β1tr-3 could be identified (Table 2). (Conversely, although there are many additional EST sequences that correspond exactly with ESTa (Table 2), we did not amplify any precisely matching sequences from zebrafish embryos.) EST sequences also provide evidence concerning the expression of the different β1 paralogs in different tissues or at different stages of development (Table 2). ESTs for the truncated paralogs (especially ESTs corresponding to ESTa) are found in cDNA from several different tissues, and appear to be particularly abundant in liver and kidney.

## Discussion

The novel findings of this report are: (i) Extensive expansion of the β1 integrin gene family has taken place in zebrafish, (ii) a group of divergent β1 paralogs with regulatory site mutations is present, some of which are novel truncated forms that lack the transmembrane and intracellular domains, (iii) the different β1 paralogs exhibit varied patterns of temporal and tissue expression. Our findings raise several important questions. For example: Why do zebrafish have multiple versions of the single β1 gene found in higher vertebrates? What is the function of the divergent paralogs? Which α subunits does each paralog pair with?

Gene duplication is an important mechanism for the generation of phenotypic complexity, diversity and innovation. Gene duplication can occur either through tandem duplication of an individual gene, segmental duplication of a portion of a chromosome, or through whole genome duplication. Recent analyses have provided strong support for a whole genome duplication in the lineage of ray-finned fishes approx. 350 million years ago [34-36]. Following this duplication, most of the gene duplicates were rapidly lost. However, a small proportion of these duplicates were retained. Hence, for approximately 20% of single-copy human genes, two corresponding orthologs are found in zebrafish [37]. It is possible that β1–1 and β1–2 arose from the fish-specific whole genome duplication. Consistent with this possibility, β1–1 and β1–2 have different chromosomal locations and furthermore, both gene loci show synteny with the p11.22 region of human chromosome 10, which contains the β1 (ITGB1) gene (APM, unpublished data). Two co-orthologs of β1 are also found in other teleost species such as fugu [38].

In the classical model of gene duplication [39], new gene duplicates face one of two fates: either one copy mutates to a pseudogene (called nonfunctionalization), or one copy preserves the original function, and the other copy mutates freely until by chance it obtains a sequence that confers a new, beneficial, positively selected function (called neofunctionalization). In a third possibility, called subfunctionalization or subfunction partitioning, the complementary partitioning of ancestral subfunctions is the mechanism that preserves gene duplicates [37]. Two classic examples of subfunction partitioning in zebrafish are the pair of sox9 genes, sox9a and sox9b [40], and the two mitf genes, mitfa and mitfb [41]. It is possible that ancestral β1 integrin functions have been partitioned between β1–1 and β1–2. Subfunction partitioning can occur through tissue-specific expression of gene duplicates and/or expression at different times of development. We found that there are differences in temporal expression of β1–1 and β1–2, with the expression of β1–2 peaking in early development, whereas expression of β1–1 was highest during later development. Also some differences in the tissue expression between these two paralogs were noted, with low expression of β1–1 in adult intestine. It is also possible that partitioning could take place if each β chain associates with a distinct subset of α chain partners. The possible subfunction partitioning of β1 roles by β1–1 and β1–2 may provide distinct advantages for functional genetic analyses in zebrafish. Whereas knockout of the single β1 gene in mice is very early embryonic lethal [9,10], knockout (or knockdown) of only one of these genes in zebrafish may have a much less severe phenotype, making it possible to study the roles of β1 integrins during the later stages of development.

In contrast to β1–1 and β1–2, it seems likely that the divergent β1 paralogs arose through tandem duplications. Taking the genomic, phylogenetic and sequence identity data together, the most likely scenario is that the precursor of β1–2 underwent a tandem duplication that gave rise to the ancestor of β1–3; a subsequent duplication of this paralog gave rise to the progenitor of the truncated paralogs. It seems likely that the divergent paralogs have acquired novel functions. With the exception of β1tr-3, these paralogs are expressed at later times of development and in a more tissue-restricted manner than β1–1 and β1–2. It therefore appears that the divergent paralogs may be involved in tissue differentiation, maintenance or remodelling, rather than in development/organogenesis. The expression of many of these paralogs in tissues such as liver and kidney supports this view.

One clue to the function of the divergent paralogs is that they possess a double substitution (DD to SN) in the regulatory ADMIDAS cation-binding site in the A-domain. Preliminary results suggest that engineering an identical substitution in human β1 (in a heterodimer with α5 subunit) produces a constitutively inactive integrin, i.e., an integrin with very low affinity for ligand (J. Askari and A.P. Mould, unpublished data). It is therefore possible that β1–3 could act as a dominant-negative β1 subunit by competing with β1–1 and β1–2 for α subunit partners or intracellular binding factors. It is also proposed that expression of high levels of unoccupied integrins can trigger apoptosis [42]; hence, expression of β1-3 could also regulate cell survival. If the truncated paralogs are unable to associate with α subunits they could be secreted into the extracellular environment. There are now many examples of cell surface receptors that also have a secreted antagonistic counterpart, e.g. VEGFR, FGFR-1 and IL-1R [43-45]. It is therefore intriguing to speculate that truncated integrin paralogs could have a similar mode of action.

It is also possible that the divergent paralogs could have acquired new binding partners. The very high degree of sequence conservation of the PSI, hybrid domain and EGF-1 between the divergent paralogs suggest that these domains form a key functional region of these polypeptides, and furthermore that the function of these domains is common to all the divergent β1 paralogs. Such a degree of conservation suggests that these domains interact with protein ligands (even though there is currently no evidence in other integrins that these domains have extracellular ligands). The PSI, hybrid, and EGF-1 domains lie adjacent to each other at, or close to, the knee region of the integrin [15]. Proteins that bind to these domains could therefore alter function, either by constraining the integrin in a bent (low affinity) form or by perturbing the interactions between PSI/hybrid/EGF-1 domains and other integrin domains, thereby favouring the unbent (high affinity) form. A key hinge point in the β subunit knee region is thought to be the near the start of EGF-2 [15]. Interestingly, some of the truncated paralogs have a six amino acid deletion in this linker region (Fig. 4), which is likely to change the conformational state of the protein, and may therefore modify function. Intriguingly, the second EGF repeat in β1tr-3 is essentially identical to that of β1–3, implying that this repeat may also have a conserved role between these two paralogs.

It is currently unclear how many separate genes are represented by the truncated β1 paralogs and ESTs. Although the sequences β1tr-1 and β1tr-2 are 98% identical to each other they are probably distinct gene products because they have different 3'UTRs. In addition, although β1tr-2 and β1tr-3 may have identical 3'UTR sequences, it appears unlikely that β1tr-3 is an alternatively spliced form of β1tr-2 because the differences between the two coding sequences are spread throughout the primary structures (Fig. 4 and Additional file 1). Additionally, the truncated paralogs cannot simply represent different alleles of the same gene because at least three of these paralogs could be amplified from RNA derived from a single fish (APM and Paul Walker, unpublished results). Furthermore, the genomic sequence that corresponds to ESTb (which is 98% identical in coding sequence to β1tr-2) does not contain sequences that would allow the generation of different transcripts by alternative splicing (data not shown). In summary, the currently available evidence suggests that the truncated paralogs originate from separate (yet to be characterised) genes. Since the genomic scaffold is assembled from shotgun sequences it is possible that, due to the almost identical sequences of the truncated paralogs, the scaffold is incorrectly assembled and additional genes of truncated paralogs may be present in the same region. In the future, the availability of finished clones in the region of the genome that contains 'ESTb' for detailed analysis should help to clarify the precise number of truncated β1 paralogs in the zebrafish genome. A further puzzle is why none of the truncated β1 sequences we amplified from zebrafish RNA exactly matched the ESTa or ESTb sequences. A possible explanation is that the β1tr-1, β1tr-2 and β1tr-3 sequences were only the predominant sequences obtained by subcloning of the RT-PCR products. Other clones obtained have not yet been fully sequenced, some of these may match the EST sequences more precisely.

Truncated forms of β subunits have previously been reported in sponge and man [7,46]. However, these polypeptides represent alternatively spliced forms of full-length β subunits that are truncated within the hybrid domain, and therefore probably result in non-functional proteins [47]. In contrast, the truncated β1 paralogs reported here appear to be unique gene products, and the position of the truncation would preserve the structure and function of all of the extracellular domains with the exception of the βTD.

A key subject of future investigations will be to identify the α subunit partners of the different paralogs. All twelve α chains that pair with β1 in higher vertebrates have orthologs in the zebrafish genome, and at least two of these α chains have two co-orthologs (APM, unpublished results). It seems very unlikely that all of the β1 paralogs identified here are able to associate with all of the >14 potential α chain partners. For example, if each of the six β1 paralogs has retained the ability to pair with all of the α chains then a minimum of 84 different heterodimers could form. This would present a huge increase in complexity over the twelve heterodimers found in higher vertebrates. It is likely, therefore that in this process of multimeric protein evolution, previously termed 'molecular incest' [48], that only a limited number of possible combinations was retained based on function.

It is not yet clear if divergent β1 paralogs are found in other teleosts. Currently only limited gene information is available on other fish species, with the exception of the pufferfish *Takifugu rubripes *and *Tetraodon nigroviridis*. Analysis of the pufferfish genomes suggests that only two β1 paralogs are present in fugu and tetraodon; these paralogs are closely related to zebrafish β1–1 and β1–2 (AP Mould, unpublished results). However, since β1–3 probably arose from an ancient duplication of the ancestor of β1–2 it would be surprising if the former paralog was retained only in the zebrafish lineage. Furthermore, although the divergent β1 paralogs have not been detected in pufferfish genomes, pufferfish may have retained fewer gene duplicates than zebrafish [49].

The presence of expanded gene families is hypothesized to have made a major contribution to the extraordinary diversity and evolutionary success of teleosts, which make up half of all vertebrate species. It is known that fish genomes are more 'plastic' than other vertebrate genomes, partly due to higher rates of gene duplication [50]. Fish-specific novel members of gene families may contribute to a large extent to the distinct physiology of fishes and mammals, while differential retention of duplicate genes may have facilitated the isolation of emerging species during the vast radiation of teleosts [51,52]. Our findings show that integrins are a dynamic family of genes that have evolved in multiple ways after the divergence of the common ancestors of the mammalian and fish lineages. Expansion of the integrin family may also correlate with expansion of extracellular matrix gene families, e.g. collagens, in teleosts [34].

## Conclusion

At least six paralogs of the integrin β1 gene have been identified in zebrafish, demonstrating that this species has a greatly expanded integrin repertoire. Two of these paralogs may share the functions of the single β1 subunit found in higher vertebrates, whereas the remaining paralogs may have acquired novel roles. This is the first description of truncated β1 chains, which we speculate could be secreted proteins that act as regulators of integrin functions.

## Methods

### Sequencing of zebrafish integrin β1 paralogs

The complete amino acid sequence of human β1 [Swiss-Prot: P05556] was used to probe the Danio rerio genome ([59,61]) using TBLASTN [53] to identify genes with highest sequence identity to human β1. To amplify complete β1 sequences, primers were designed using putative 5' and 3' sequences (identified as described above) or from EST sequences on the NCBI database [60]. The following primers were used: β1–1, 5'-ATGGACCTGAAGCTACTTTTCATATC-3' and 5'-CTGATGGCCATTATTTGCCTTCG-3', β1–2, 5'-ATGGACGTAAGGCTGCTCCTG-3'and 5'-CACGTTCGTCCATTATTTGCCCTC-3', β1–3, 5'-ATGAAAATGAAGCTGCTGTTATTATC-3' and 5'-CACTTTCCCTCATATCTGGGATTC-3' β1tr-1, 5'-ATGGATATAACAGTTTTGTTATTATCAG-3' and 5'-ATGTATAACATGAGGTCATGATGTAC-3' β1tr-2 5'-ATGGATATAACAGTTTTGTTATTATCAG-3' and 5'-GTATAACATGTGTCTCAATATATGATG-3'

Total RNA was prepared from 4-day old embryos using TRI reagent (Sigma), and reverse transcription was performed using Superscript II (Invitrogen) according to the manufacturer's instructions. PCR reactions were performed using Phusion (New England Biolabs). Cycling parameters were 98°C for 30 s, followed by 40 cycles of 98°C for 10 s, 60°C for 20 s and 72°C for 60 s. An additional sequence, β1tr-3, was amplified from reverse transcribed RNA from adult kidney (prepared as described below) using the same primers as for β1tr-2. The PCR reactions generated products of ~2.4 kB for β1–1, β1–2 and β1–3, and ~1.9 kB for β1tr-1, β1tr-2 and β1tr-3. EST clones ESTa and ESTb were obtained from RZPD German Resource Center for Genome Research, product numbers IRAKp961B08165Q [IMAGE:6525557] from adult kidney cDNA library and IMAGp998I1214695Q3 [IMAGE:7001749] from whole adult body cDNA library, respectively.

PCR products were analysed on 1.5% agarose gels. Sequencing of PCR products or EST clones was performed using the BigDye cycle sequencing kit (Applied Biosystems). PCR products from reactions using the 5'-ATGGATATAACAGTTTTGTTATTATCAG-3' forward primer contained a mixture of sequences and therefore these products were subcloned into the Zero Blunt TOPO cloning kit (Invitrogen). Sequencing of individual clones revealed a single most abundant sequence (β1tr-1, β1tr-2 or β1tr-3). Alignment of zebrafish and human sequences was performed using CLUSTAL W [62].

### Phylogenetic analysis

The β1 integrin sequences identified in *Danio rerio *were aligned with the human β1 sequence [Swiss-Prot: P05556] and the ancestral β1-like gene previously identified in *Ciona intestinalis *(JGI Ciona v1.0 ci0100141446) [8] using CLUSTAL X [54]. Gap-containing sites were removed from each alignment and Maximum Likelihood trees were inferred using PROML from the PHYLIP package [55]. The JTT model of amino acid substitutions was used with and without global rearrangements and correction for rate heterogeneity (α value obtained from TREEPUZZLE [56]). The topologies of the trees were tested using three independent methods. Neighbor-Joining and Maximum Parsimony bootstrap replicates were obtained using the PHYLIP package [55]. Bayesian tree inference values were produced from the MrBayes programme [57].

### RT-PCR analysis

Embryos were harvested at different times post fertilization. Tissues from adult fish (~9 months old) were removed by dissection and flash frozen in liquid nitrogen. Total RNA was prepared using Trizol (QIAGEN). Reverse transcription was performed using M-MLV reverse transcriptase or Omniscript (Invitrogen) according to the manufacturer's instructions. PCR reactions were performed using recombinant Taq (a gift from P. Walker, University of Manchester, UK) or Advantage 2 polymerase (BD Biosciences). For the truncated β1 sequences, primers were designed to give a single product of approx. 600 bp that is unique to β1tr-1, β1tr-2 or β1tr-3. The following primers were used:

β1–1 5'-ATGGACCTGAAGCTACTTTTCATATC-3' and 5'-GTGACGTTTCTCCAGCCAATGTG β1–2 5'-GATGGTAATGAATGCACCAAGGC-3' and 5'-GGAGTCGGAGGTAAGCGTTCC-3' β1–3 5'-GTGTTGTTTGATATAGAAATCACGGCT-3' and 5'-CGTATCCCACTTGGCATTATTTTTCTC-3' β1tr-1 5'-CCAGGATCTGGATGCATACTG-3' and 5'-ATGTATAACATGAGGTCATGATGTAC-3' β1tr-2 5'-CCAGGATCTGGATGCATACTG-3' and 5'-GTATAACATGTGTCTCAATATATGATG-3' β1tr-3 5'-CATGATGAGGTGCTGGCGGATG-3' and 5'-GTATAACATGTGTCTCAATATATGATG-3'

Cycling parameters were 95°C for 2 min, followed by 30 cycles of 95°C for 30 s, 60°C for 20 s and 72°C for 60 s. PCR products were analysed on 1.5% agarose gels. The identity of selected products was confirmed by DNA sequencing. As a control, a fragment of β-actin was amplified using the primers 5'-CCACGAGACCACCTTCAACT-3' and 5'-CATTGTGAGGAGGGCAAAGT-3' for 28 cycles of 95°C for 30 s, 55°C for 40 s and 72°C for 60 s. Negative controls consisted of no cDNA template reactions.

## Authors' contributions

APM and MJH conceived of the study. APM and JAM carried out the database searching, PCR reactions and sequence analysis, JHJ performed the phylogenetic analyses and ACG prepared RNA for RT-PCR analysis, APM and ACG carried out the RT-PCR analysis. All authors participated in the interpretation of data and/or in the writing of the manuscript.

## Supplementary Material

Additional File 1 DNA sequence alignment of truncated β1 paralogs. TGA stop codon is shown in bold font. The portion of the 3'UTR used to design reverse primers is shown boxed. PolyA tail region is shown underlined. Sequence identities are indicated by *. Click here for file

Additional File 2 Protein sequence alignment of zebrafish β1 sequences with human and Ciona β1 orthologs. Alignment was performed using Clustal X and is displayed using Boxshader. Levels of sequence conservation are indicated (>50% identical, red; conservative substitutions, blue). Note that the refined Ciona β1 sequence [8] is missing the signal peptide, the PSI domain and part of the hybrid domain. Click here for file

## References

[B1] Hynes RO (2002). Integrins: Bidirectional, allosteric signaling machines. Cell.

[B2] DeMali KA, Wennerberg K, Burridge K (2003). Integrin signaling to the actin cytoskeleton. Curr Opin Cell Biol.

[B3] Schwartz MA, Assoian RK (2001). Integrins and cell proliferation: regulation of cyclin-dependent kinases via cytoplasmic-signalling pathways. J Cell Sci.

[B4] Ginsberg MH, Partridge A, Shattil SJ (2005). Integrin regulation. Curr Opin Cell Biol.

[B5] Mould AP, Humphries MJ (2004). Regulation of integrin function through conformational complexity: not simply a knee-jerk reaction?. Curr Opin Cell Biol.

[B6] Springer TA, Wang JH (2004). The three-dimensional structure of integrins and their ligands, and conformational regulation of cell adhesion. Adv Protein Chem.

[B7] Brower DL, Brower SM, Hayward DC, Ball EE (1997). Molecular evolution of integrins: Genes encoding integrin β subunits from a coral and a sponge. Proc Nat Acad Sci USA.

[B8] Ewan R, Huxley-Jones J, Mould AP, Humphries MJ, Robertson DL, Boot-Handford RP (2005). The integrins of the urochordate *Ciona intestinalis *provide novel insights into the molecular evolution of the vertebrate integrin family. BMC Evol Biol.

[B9] Fassler R, Meyer M (1995). Consequences of lack of β1 integrin gene expression in mice. Genes Dev.

[B10] Stephens LE, Sutherland AE, Klimanskaya IV, Andrieux A, Meneses J, Pedersen RA, Damsky CH (1995). Deletion of β1 integrins in mice results in inner cell mass failure and peri-implantation lethality. Genes Dev.

[B11] Brakebusch C, Fässler R (2005). β1 integrin function in vivo: adhesion, migration and more. Cancer Metastasis Rev.

[B12] Danen EHJ, Sonnenberg A (2003). Integrins in regulation of tissue development and function. J Path.

[B13] Xiong JP, Stehle T, Diefenbach B, Zhang R, Dunker R, Scott DL, Joachimiak A, Goodman SL, Arnaout MA (2001). Crystal structure of the extracellular segment of integrin αVβ3. Science.

[B14] Xiao T, Takagi J, Coller BS, Wang JH, Springer TA (2004). Structural basis for allostery in integrins and binding to fibrinogen-mimetic therapeutics. Nature.

[B15] Shi M, Sundramurthy K, Liu B, Tan SM, Law SK, Lescar J (2005). The crystal structure of the plexin-semaphorin-integrin domain/hybrid domain/I-EGF1 segment from the human integrin β2 subunit at 1.8Å resolution. J Biol Chem.

[B16] Tuckwell DS, Humphries MJ (1997). A structure prediction for the ligand-binding region of the integrin β subunit: evidence for the presence of a von Willebrand factor A domain. FEBS Lett.

[B17] Takagi J, Kamata T, Meredith J, Puzon-McLaughlin W, Takada Y (1997). Changing ligand specificities of αvβ1 and αvβ3 integrins by swapping a short diverse sequence of the β subunit. J Biol Chem.

[B18] Lin EC, Ratnikov BI, Tsai PM, Carron CP, Myers DM, Barbas CF, Smith JW (1997). Identification of a region in the integrin β3 subunit that confers ligand binding specificity. J Biol Chem.

[B19] Takagi J, DeBottis DP, Erickson HP, Springer TA (2002). The role of the specificity-determining loop of the integrin β subunit I-like domain in autonomous expression, association with the α subunit, and ligand binding. Biochemistry.

[B20] Mould AP, Barton SJ, Askari JA, Craig SE, Humphries MJ (2003). Role of ADMIDAS cation-binding sites in ligand recognition by integrin α5β1. J Biol Chem.

[B21] Chen J, Salas A, Springer TA (2003). Bistable regulation of integrin adhesiveness by a bipolar metal ion cluster. Nat Struct Biol.

[B22] Mould AP, Barton SJ, Askari JA, McEwan PA, Buckley PA, Craig SE, Humphries MJ (2003). Conformational changes in the integrin β A domain provide a mechanism for signal transduction via hybrid domain movement. J Biol Chem.

[B23] Hughes PE, Diaz-Gonzalez F, Leong L, Wu C, McDonald JA, Shattil SJ, Ginsberg MH (1996). Breaking the integrin hinge. A defined structural constraint regulates integrin signaling. J Biol Chem.

[B24] Kiema T, Lad Y, Jiang P, Oxley CL, Baldassarre M, Wegener KL, Campbell ID, Ylanne J, Calderwood DA (2006). The molecular basis of filamin binding to integrins and competition with talin. Mol Cell.

[B25] Nasevicius A, Ekker SC (2000). Effective targeted gene 'knockdown' in zebrafish. Nat Genet.

[B26] Wienholds E, Schulte-Merker S, Walderich, Plasterk RH (2002). Target-selected inactivation of the zebrafish rag1 gene. Science.

[B27] Berghmans S, Jette C, Langenau D, Hsu K, Stewart R, Look T, Kanki JP (2005). Making waves in cancer research: new models in the zebrafish. Biotechnique.

[B28] Zon LI, Peterson RT (2005). In vivo drug discovery in the zebrafish. Nat Rev Drug Discov.

[B29] Wehrle-Haller B, Imhof BA (2003). Integrin-dependent pathologies. J Pathol.

[B30] Sheppard D (2000). In vivo functions of integrins: lessons from null mutations in mice. Matrix Biol.

[B31] Bouvard D, Brakebusch C, Gustafsson E, Aszodi A, Bengtsson T, Berna A, Fassler R (2001). Functional consequences of integrin gene mutations in mice. Circ Res.

[B32] Qian Y, Noya M, Ainsworth AJ (2000). Molecular characterization and leukocyte distribution of a teleost β1 integrin molecule. Vet Immunol Immunopathol.

[B33] Yamanouchi J, Hato T, Tamura T, Fujita S (2002). Identification of critical residues for ligand binding in the integrin β3 I-domain by site-directed mutagenesis. Thromb Haemost.

[B34] Jaillon O, Aury JM, Brunet F, Petit JL, Stange-Thomann N, Mauceli E, Bouneau L, Fischer C, Ozouf-Costaz C, Bernot A, Nicaud S, Jaffe D, Fisher S, Lutfalla G, Dossat C, Segurens B, Dasilva C, Salanoubat M, Levy M, Boudet N, Castellano S, Anthouard V, Jubin C, Castelli V, Katinka M, Vacherie B, Biemont C, Skalli Z, Cattolico L, Poulain J, De Berardinis V, Cruaud C, Duprat S, Brottier P, Coutanceau JP, Gouzy J, Parra G, Lardier G, Chapple C, McKernan KJ, McEwan P, Bosak S, Kellis M, Volff JN, Guigo R, Zody MC, Mesirov J, Lindblad-Toh K, Birren B, Nusbaum C, Kahn D, Robinson-Rechavi M, Laudet V, Schachter V, Quetier F, Saurin W, Scarpelli C, Wincker P, Lander ES, Weissenbach J, Roest Crollius H (2004). Genome duplication in the teleost fish Tetraodon nigroviridis reveals the early vertebrate proto-karyotype. Nature.

[B35] Meyer A, Van de Peer Y (2005). From 2R to 3R: evidence for a fish-specific genome duplication (FSGD). Bioessays.

[B36] Christoffels A, Koh EG, Chia JM, Brenner S, Aparicio S, Venkatesh B (2004). Fugu genome analysis provides evidence for a whole-genome duplication early during the evolution of ray-finned fishes. Mol Biol Evol.

[B37] Postlethwait J, Amores A, Cresko W, Singer A, Yan Y-L (2004). Subfunction partitioning, the teleost radiation and the annotation of the human genome. Trends Genet.

[B38] Huhtala M, Heino J, Casciari D, de Luise A, Johnson MS (2005). Integrin evolution: insights from ascidian and teleost fish genomes. Matrix Biol.

[B39] Ohno S (1970). Evolution by gene duplication.

[B40] Yan YL, Willoughby J, Liu D, Crump JG, Wilson C, Miller CT, Singer A, Kimmel C, Westerfield M, Postlethwait JH (2005). A pair of Sox: distinct and overlapping functions of zebrafish sox9 co-orthologs in craniofacial and pectoral fin development. Development.

[B41] Lister JA, Close J, Raible DW (2001). Duplicate mitf genes in zebrafish: Complementary expression and conservation of melanogenic potential. Dev Biol.

[B42] Stupack DG, Puente XS, Boutsaboualoy S, Storgard CM, Cheresh DA (2001). Apoptosis of adherent cells by recruitment of caspase-8 to unligated integrins. J Cell Biol.

[B43] Kendall RL, Wang G, Thomas KA (1996). Identification of a natural soluble form of the vascular endothelial growth factor receptor, FLT-1, and its heterodimerization with KDR. Biochem Biophys Res Commun.

[B44] Root LL, Shippley GD (2000). Normal human fibroblasts produce membrane-bound and soluble isoforms of FGFR-1. Mol Cell Biol Res Commun.

[B45] Arend WP (1991). Interleukin 1 receptor antagonist. A new member of the interleukin 1 family. J Clin Invest.

[B46] Djaffar I, Chen YP, Creminon C, Maclouf J, Cieutat AM, Gayet O, Rosa JP (1994). A new alternative transcript encodes a 60 kDa truncated form of integrin β3. Biochem J.

[B47] Coe AP, Askari JA, Kline AD, Robinson MK, Kirby H, Stephens PE, Humphries MJ (2001). Generation of a minimal α5β1 integrin-Fc fragment. J Biol Chem.

[B48] Boot-Handford RP, Tuckwell DS (2003). Fibrillar collagen: the key to vertebrate evolution? A tale of molecular incest. Bioessays.

[B49] Taylor JS, Braasch I, Frickey T, Meyer A, Van de Peer Y (2003). Genome duplication, a trait shared by 22000 species of ray-finned fish. Genome Res.

[B50] Venkatesh B (2003). Evolution and diversity of fish genomes. Curr Opin Genet Dev.

[B51] Loh YH, Christoffels A, Brenner S, Hunziker W, Venkatesh B (2004). Extensive expansion of the claudin gene family in the teleost fish, *Fugu rubripes*. Genome Res.

[B52] Steinke D, Salzburger W, Braasch I, Meyer A (2006). Many genes in fish have species-specific asymmetric rates of molecular evolution. BMC Genomics.

[B53] Altschul SF, Madden T, Schaffer A, Zhang J, Zhang Z, Miller W, Lipman DJ (1997). Gapped BLAST and PSI-BLAST: a new generation of protein database search programs. Nucleic Acid Res.

[B54] Thompson JD, Gibson TJ, Plewniak F, Jeanmougin F, Higgins DG (1997). The ClustalX windows interface: flexible strategies for multiple sequence alignment aided by quality analysis tools. Nucleic Acids Research.

[B55] Felsenstein J (1993). PHYLIP (Phylogenetic Analysis Using Parsimony). Distributed by the author: Department of Genetics, University of Washington, Seattle, USA.

[B56] Strimmer K, von Haesler A (1996). Likelihood-mapping: a simple method to visual phylogenetic content of a sequence alignment. Proc Nat Acad Sci USA.

[B57] Huelsenbeck JP (2000). MrBayes: Bayesian inference of phylogeny. Distributed by the author: Department of Biology, University of Rochester, USA.

[B58] Takagi J, Beglova N, Yalamanchili P, Blacklow SC, Springer TA (2001). Definition of EGF-like, closely interacting modules that bear activation epitopes in integrin β subunits. Proc Natl Acad Sci USA.

[B59] http://www.ensembl.org/Danio_rerio/index.html.

[B60] http://www.ncbi.nlm.nih.gov.

[B61] http://www.sanger.ac.uk/cgi-bin/blast/submitblast/d_rerio.

[B62] http://www.ebi.ac.uk/clustalw.

