# Supplementary material for: Identification of multiple integrin β1 homologs in zebrafish (Danio rerio)
Source: BMC Cell Biol. 2006 Jun 20;7:24. doi: 10.1186/1471-2121-7-24 (PMC1538996; doi:10.1186/1471-2121-7-24)
Supplement: Additional File 1 — DNA sequence alignment of truncated β1 paralogs. TGA stop codon is shown in bold font. The portion of the 3'UTR used to design reverse primers is shown boxed. PolyA tail region is shown underlined. Sequence identities are indicated by *. [file 1471-2121-7-24-S1.doc]

Additional File 1

tr-1 ATGGATATAACAGTTTTGTTATTATCAGCTCTGCTAGGATTTGTTTCTGATGTCGGTGCT 60

tr-2 ATGGATATAACAGTTTTGTTATTATCAGCTCTGCTAGGATTTGTTTCTGATGTCGGTGCT 60

tr-3 ATGGATATAACAGTTTTGTTATTATCAGCTCTGCTAGGATTTGTTTCTGATGTCGGTGCT 60

ESTa ATGGATATAACAGTTTTGTTATTATCAGCTCTGCTAGGATTTGTTTCTGATGTCGGTGCT 60

ESTb ATGGATATAACAGTTTTGTTATTATCAGCTCTGCTAGGATTTGTTTCTTATGTCGGTGCT 60

************************************************ ***********

tr-1 AACACAGACAGTAATCCATGCATTTCTGCAAATGCCAAAACCTGTGGAGAGTGTATTCAG 120

tr-2 AACACAGACAGTAATCCATGCATTTCTGCAAATGCCAAAACCTGTGGAGAGTGTATTCAG 120

tr-3 AACACAGACAGTAATCCATGCATTTCTGCAAATGCCAAAACCTGTGGAGAGTGTATTCAG 120

ESTa AACACAGACAGTAATCCATGCATTTCTGCAAATGCCAAAACCTGTGGAGAGTGTATTCAG 120

ESTb AACACAGACAGTAATCCATGCATTTCTGCAAATGCCAAAACCTGTGGAGAGTGTATTCAG 120

************************************************************

tr-1 ATCGGCCCACAGTGTGTGTGGTGCAAAGATCCTGATTTCAAACCTTCACGCTGTGATGAC 180

tr-2 ATCGGCCCACAGTGTGTGTGGTGCAAAGATCCTGATTTCAAACCTTCACGCTGTGATGAC 180

tr-3 ATCGGCCCACAGTGTGTGTGGTGCAAAGATCCTGATTTCAAACCTTCACGCTGTGATGAC 180

ESTa ATCGGCCCACAGTGTGTGTGGTGCAAAGATCCTGATTTCAAACCTTCACGCTGTGATGAC 180

ESTb ATCGGCCCACAGTGTGTGTGGTGCAAAGATCCTGATTTCAAACCTTCACGCTGTGATGAC 180

************************************************************

tr-1 ATTGAGTCCATGGCGAAAGCTGGCTGCACTGCAGACGGTGTAGAAAACCCTCGAGGAGCG 240

tr-2 ATTGAGTCCATGGCGAAAGCTGGCTGCACTGCAGACGGTGTAGAAAACCCTCGAGGAGCG 240

tr-3 ATTGAGTCCATGGCGGAAGCTGGCTGCACTGCAGACGGTGTAGAAAACCCTCGAGGAGCG 240

ESTa ATTGAGTCCATGGCGAAAGCTGGCTGCACTGCAGACGGTGTAGAAAACCCTCGAGGAGCG 240

ESTb ATTGAGTCCATGGCGAAAGCTGGCTGCACTGCAGACGGTGTAGAAAACCCTCGAGGAGCG 240

*************** ********************************************

tr-1 GTCACTATTGACAAGAACAAACCCGTCACAAACCGTAAAACTGATGGAGGACAGAATCTG 300

tr-2 GTCACTATTGACAAGAACAAACCCGTCACAAACTGTAAAATTGATGGAGGACAGAATCTG 300

tr-3 GTCACTATTGACAAGAACAAACCCGTCACGAACCGTAAAACTGATGGAGGACAGAATCTG 300

ESTa GTCACTATTGACAAGAACAAACCCGTCACAAACCGTAAAACTGATGGAGGACAGAATCTG 300

ESTb GTCACTATTGACAAGAACAAACCCGTCACAAACCGTAAAACTGATGGAGGACAGAATCTG 300

***************************** *** ****** *******************

tr-1 AGACCCGATGAGATAACGCAGATTCAGCCACAGAAAGTCACGCTGAACCTTCGCTCAGGA 360

tr-2 AGGCCTGATGAGATCACGCAGATTCAGCCACAGAAAGTCACGCTGAACCTTCGCTCAGGA 360

tr-3 AGGCCTGATGAGATCACGCAGATTCAGCCACAGAAAGTCACGCTGAACCTTCGCTCAGGA 360

ESTa AGGCCCGATGAAATCACGCAGATTCAGCCACAGAAAGTCAAGCTGAACCTTCGCTCAGGA 360

ESTb AGGCCCGATGAAATCACGCAGATTCAGCCACAGAAAGTCAAGCTGAACCTTCGCTCAGGA 360

** ** ***** ** ************************* *******************

tr-1 GAAGCACAGAAGTTTACTCTCAAGTTTAAGAGGGCAGAGGATTATCCCATCGATCTGTAC 420

tr-2 GAAGCACAGAAGTTTACTCTCAAGTTTAAGAGGGCAGAGGATTATCCCATCGATCTGTAC 420

tr-3 GAAGCACAGAAGTTTACTCTCAAGTTTAAGAGGGCAGAGGATTATCCCATCGATCTGTAC 420

ESTa GAAGCACAGAAGTTTACTCTCAAGTTTAAGAGGGCAGAGGATTATCCCATTGATCTGTAC 420

ESTb GAAGCACAGAAGTTTACTCTCAAGTTTAAGAGGGCAGAGGATTATCCCATTGATCTGTAC 420

************************************************** *********

tr-1 TTCCTGATGGACCTCAGTCACTCTATGCTAAGCAATTTGGAAAATTTAAAAAACCTGGGA 480

tr-2 TTCCTGATGGACCTCAGTCACTCTATGCTGAGCAATTTGGAAAATTTCAAAAACCTGGGA 480

tr-3 TTCCTGATGGACCTCAGTCACTCTATGCTGAGCAATTTGGAAAATTTAAAAAACCTGGGA 480

ESTa TTCCTGATGGACCTCAGTCACTCTATGCTGAGCAATTTGGAAAATTTCAAAAACCTGGGA 480

ESTb TTCCTGATGGACCTCAGTCACTCTATGCTGAGCAATTTGGAAAATTTCAAAAACCTGGGA 480

***************************** ***************** ************

tr-1 TTCGAACTCGCCAAAGAGATGAAGGACATCACGAAAGACCTGCGTATAGGTTTTGGTTCA 540

tr-2 ACCGAACTCGCCAATGAGATGAAGGACATCACGAAAGACCTGCGTATAGGTTTTGGTTCA 540

tr-3 ACCGAACTCGCCAATGAGATGAAGGACATCACGAAAGACCTGCGTATAGGTTTTGGTTCA 540

ESTa ACCGAACTCGCCAATGAGATGAAGGACATCACGAAAGACCTGCGTATAGGTTTTGGTTCA 540

ESTb ACCGAACTCGCCAATGAGATGAAGGACATCACGAAAGACCTGCGTATAGGTTTTGGTTCA 540

************ *********************************************

tr-1 TTCTTTAGGAAACCTTCCATTCAGACGAACCCATGTTTTCCAGATAATTGCATAGCTCCA 600

tr-2 TTCTTTAGGAAACCTTCCATTCAGACGAACCCATGTTTTCCAGATAATTGCATAGCTCCA 600

tr-3 TTCTTTAGGAAACCTTCCATTCAGACGAACCCATGTTTTCCAGATAATTGCATAGCTCCA 600

ESTa TTCTTTAGGAAACCTTCCATTCAGACGAACCCATGTTTTCCAGATAATTGCATAGCTCCA 600

ESTb TTCTTTAGGAAACCTTCCATTCAGACGAACCCATGTTTTCCAGATAATTGCATAGCTCCA 600

************************************************************

tr-1 TTCAGTTACTTTAATGTACTGAGCTTGACGGACGATCATGCATTGTTCACACAAGAAATC 660

tr-2 TTCAGTTACTTTAATGTACTGAGCTTGACGGACGATCATGCATTGTTTACACAAGAAATC 660

tr-3 TTCAGTTACTTTAATGTACTGAGCTTGACGGACGATCATGCATTGTTTACTCAAGAAATC 660

ESTa TTCAGTTACTTTAATGTACTGAGCTTGACGGACGATCATGCATTGTTTACACAAGAAATC 660

ESTb TTCAGTTACTTTAATGTACTGAGCTTGACGGACGATCATGCATTGTTTACACAAGAAATC 660

*********************************************** ** *********

tr-1 AGCAAGCTGAAAACATCTGGAAACCTGGATTCTTCAGAGGCAGGATTAGAGGCATTGATG 720

tr-2 AGCAAGCTGAAAACATCTGGAAACCTGGATTCTTCAGAGGCAGGATTAGAGGCATTGATG 720

tr-3 AGCAAGCTGAAAACCTCTGGAAACCTGGATTCTTCAGAGGCAGGATTAGAGGCATTGATG 720

ESTa AGCAAGCTGAAAACCTCTGGAAACCTGGATTCTTCAGAGGCAGGATTAGAGGCATTGATG 720

ESTb AGCAAGCTGAAAACCTCTGGAAACCTGGATTCTTCAGAGGCAGGATTAGAGGCATTGATG 720

************** *********************************************

tr-1 CAGGCTGCTGTCTGCACGGACGTGATTGGCTGGAGGAATGCCACCCGTGTCCTTGTGTTT 780

tr-2 CATGCTGCTGTCTGCACGGACGTGATTGGCTGGAGGAATGTCACCCGTGTCCTTGTGTTT 780

tr-3 CAGGCTGCTGTCTGCACGGACGTGATTGGCTGGAGGAATGTCACTCGTGTCCTTGTGTTT 780

ESTa CAGGCTGCTGTCTGCACGGACGTGATTGGCTGGAGGAATGCCACCCGTGTCCTTGTGTTT 780

ESTb CAGGCTGCTGTCTGCACGGACGTGATTGGCTGGAGGAATGTCACTCGTCTCCTTGTGTTT 780

** ************************************* *** *** ***********

tr-1 TTCACGGATGCTGGACTGCGTTTTTCTGGAGATGGAAAACGAGGTGGCATTGTTCGTCTA 840

tr-2 TTCACGGATGCTGGACTGCGTTTTTCTGGAGATGGAAAACGAGGTGGCATTGTTCGTCTA 840

tr-3 ATCACGGATGCTGGACTGCGTTTTTCTAGAGATGGAAAACGAGGTGACATTGTTCGTCTA 840

ESTa TTCACGGATGCTGGACTGCGTTTTTCTGGTGATGGAAAACGAGGTGGCATTGTTCATCTA 840

ESTb TTCACGGATGCTGGACTGCGTTTTTCTGGTGATGGAAAACGAGGTGGCATTGTTCGTCTA 840

************************** * **************** ******** ****

tr-1 AATGATGGGAAATGCCTTCTTGACGATAATATGTACACCAGAAGTGACTACTCTGACTAC 900

tr-2 AATGATGGGAAATGCCTTCTTGAGGATAATATGTACACCAGAAGTGACTACTCTGACTAC 900

tr-3 AATGATGGGAAATGCCTTCTTGACGATAATATGTACACCAGAAGTGACTACTCTGACTAC 900

ESTa AATGATGGGAAATGCCTTCTTGATGATAATATGTACACCAGAAGTGACTACTCTGACTAC 900

ESTb AATGATGGGAAATGCCTTCTTGACGATAATATGTACACCAGAAGTGACTACTCTGACTAC 900

*********************** ************************************

tr-1 CCCAGCCTCTCTCAGCTGGTAGACACAGTTACTGACAATAGCATTCACACTATCTTTGCT 960

tr-2 CCCAGCCTCTCTCAGCTGGTAGACACAGTTACTGACAATAGCATTCACACTATCTTTGCT 960

tr-3 CCCAGCCTCTCTCAGCTGGTAGACACAGTTACTGACAATAGCATTCACACTATCTTTGCT 960

ESTa CCCAGCCTCTCTCAGCTGGTAGACACAGTTACTGACAATAGCATTTACACTATCTTTGCT 960

ESTb CCCAGCCTCTCTCAGCTGGTAGACACAGTTACTGACAATAGCATTCACACTATCTTTGCT 960

********************************************* **************

tr-1 GTGACAGAGCAGTTTCAGGATCTTTATCAGGAGCTGTCTGCTAAAGTGCCTAACTCAGCA 1020

tr-2 GTGACAGAGCAGTTTCGGGATCTTTATCAGGAGCTGTCTGCTAAAGTGCCTAACTCAGCA 1020

tr-3 GTGACAGAGCAGTTTCAGGATCTTTATCAGGAGCTGTCTGCTAAAGTGCCTAACTCAGCA 1020

ESTa GTGACAGAGCAGTTTCGGGATCTTTATCAGGAGCTGTCTGCTAAAGTGCCTAACTCAGCA 1020

ESTb GTGACAGAGCAGTATCGGGATCTTTATCAGGAGCTGTCTGCTAAAGTGCCTAACTCAGCA 1020

************* ** *******************************************

tr-1 GTGGGGACATTCTCCACCAGTGGGGACAATCTGGCTAAGCTCGTCATTGATGCATTAATT 1080

tr-2 GTGGGGACATTCTCCACCAGTGGGGACAATCTGGCTAAGCTCGTCATTGATGCATTAATT 1080

tr-3 GTGGGGACACTCTCCACCAGTGGGGACAATCTGGCTAAGCTCGTCATTGATGCATTAATT 1080

ESTa GTGGGGACACTCTCCACCAGTGGGGACAATCTGGCTAAGCTCGTCATTGATGCATTAATT 1080

ESTb GTGGGGACACTCTCCACCAGTGGGGACAATCTGGCTAAGCTCGTCATTGATGCATTAATT 1080

********* **************************************************

tr-1 CCTCTGTCCTCTGAGGTGATTGTGGAGAACAGCAAGCTACCTGATGGTGTGTCCATCTCC 1140

tr-2 CCTCTGTCCTCTGAGGTGATTGTGGAGAACAGCAAGCTACCTGATGGTGTGTCCATCTCC 1140

tr-3 TCTCTGTCCTCTAAGGTGATTGTGGAGAACAGCAAGCTACCTGATGGTGTGTCCATCTCC 1140

ESTa CCTCTGTCCTCTGAGGTGATTGTGGAGAACAGCAAGCTACCTGATGGTGTGTCCATCTCT 1140

ESTb CCTCTGTCCTCTGAGGTGATTGTGGAGAACAGCAAGCTACCTGATGGTGTGTCCATCTCT 1140

*********** **********************************************

tr-1 TACGTCTCCCACTGCAAGAACGGAGTGAATGGAAGAGGAGAAGATGGGAGAAAGTGCTCC 1200

tr-2 TACGTCTCCCACTGCAAGAACGGAGTGAATGGAAGAGGAGAAGATGGGAGAAAGTGCTCC 1200

tr-3 TACGTCTCCCACTGCAAGAACGGAGTGAATGGAAGAGGAGAAGATGGGAGAAAGTGCTCC 1200

ESTa TACGTCTCCCACTGCAAGAACGGAGTGAATGGAAGAGGAGAAGATGGGAGAAAGTGCTCC 1200

ESTb TACGTCTCCCACTGCAAGAACGGAGTGAATGGAAGAGGAGAAGATGGGAGAAAGTGCTCC 1200

************************************************************

tr-1 AACATCTCCATTGGGGACGAGGTGTTGTTTGATATAGAAATCACAGCTAAAGGCTGTCCA 1260

tr-2 AACATCTCCATTGGGGACGAGGTGTTATTTGATATAGAAATCACAGCTAAAGGCTGTCCA 1260

tr-3 AGTATCTCTATTGGGGACGAGGTGTTGTTTGATATAGAAATCACGGCTAAAGGTTGTCCA 1260

ESTa AACATCTCCATTGGGGACGAGGTGTTGTTTGATATAGAAATCACGGCTAAAGGCTGTCCG 1260

ESTb AACATCTCCATTGGGGATGAGGTGTTGTTTGATATAGAAATCACGGCTAAAGGCTGTCCG 1260

* ***** ******** ******** ***************** ******** *****

tr-1 TCTAAAGGTAAACCAGAGACCATAAAGATCAAGCCGCTGGGGTTCAGCGAGGAGGTGGAG 1320

tr-2 TCTAAAGGTAAACCAGAGACCATAAAGATCAAGCCGCTGGGGTTCAGCGAGGAGGTGGAG 1320

tr-3 TCTAAAGGTAAACCAGAGACCATAAAGATCAAGCTGCTGGGGTTCAGTGAGGAGGTGGAG 1320

ESTa TCTAAAGGTAAACCAGAGACCATAAAGATCAAGCCGCTGGGGCTCAGCGAGGAGGTGGAG 1320

ESTb TCTAAAGGTAAACCAGAGACCATAAAGATCAAGCCACTGGGGTTCAGCGAGGAGGTGGAG 1320

********************************** ****** **** ************

tr-1 ATCCTCCTCAACTACATCTGTGAATGTGAGTGTCACAAAGGCGGGATCAAAAACAGTCCC 1380

tr-2 ATCCTCCTCAACTACATCTGTGAATGTGAGTGTCACAAAGACGGGATCAAAAACAGCCCC 1380

tr-3 ATCCTCCTCAACTACATCTGTGAATGTGAGTGTCACAAAGACGGGATCAAAAACAGCCCC 1380

ESTa ATCTTTCTCAACTACATCTGTGAATGTGAGTGTCACAAAGACGGGATCAAAAACAGTCCC 1380

ESTb ATCTTTCTCAACTACATCTGTGAATGTGAGTGTCACAAAGACGGGATCAAAAACAGCCCC 1380

*** * ********************************** *************** ***

tr-1 GAGTGTAGCGGAGGACAGGGAACGCTGGAGTGTGGAGTCTGCAGGTGTAATGAAGGTCGA 1440

tr-2 AAGTGTAGCGGAGGACAGGGAACGCTGGAGTGTGGAGTCTGCAGGTGTAATGAAGGTCGA 1440

tr-3 GAGTGTAGCGGAGGACAGGGAACGCTGGAGTGTGGAGTCTGCAGGTGTAATGAAGGTCGA 1440

ESTa AAGTGTAGCGGAGGACAGGGAACGCTGGAGTGTGGAGTCTGCAGGTGTAATGAAGGTCGA 1440

ESTb GAGTGTAGCGGAGGACAGGGAACGCTGGAGTGTGGAGTCTGCAGGTGTAATGAAGGTCGA 1440

***********************************************************

tr-1 TCTGGCAGAATTTGTGAGTGCACCCAGGAT------------------CTGGATGCATAC 1482

tr-2 TCTGGCAGAATTTGTGAGTGCACCCAGGAT------------------CTGGATGCATAC 1482

tr-3 TTAGGCAGATTATGTGAGTGTAGCCATGATGAGGTGCTGGCGGATGATCTGGATGCATAC 1500

ESTa TCTGGCAGATTATGTGAGTGTAGCCATGATAAGTTGCTGGCGGATGATCTGGATGCATAC 1500

ESTb TCTGGCAGAATTTGTGAGTGCACCCAGGAT------------------CTGGATGCATAC 1482

* ****** * ******** * *** *** ************

tr-1 TGCCAGATGGATATGTCGTCAGGCATCTGCAGCAACAATGGAGAATGTGTCTGTGGAACC 1542

tr-2 TGCCAGATGGATATGTCGTCAGGCATCTGCAGCAACAATGGAGAATGTGTCTGTGGAACG 1542

tr-3 TGCCGTATGAATAATGGCACAGAAGTCTGCAGCAACAATGGCGAATGTGTCTGTGGAACG 1560

ESTa TGCCGTATGAATAATGGCACAGAAGTCTGCAGCAACAATGGAGAATGTGTCTGTGGAATC 1560

ESTb TGCCAGATGGATATGTCGTCAGGCATCTGCAGCAACAATGGCGAATGTGTCTGTGGAACC 1542

**** *** *** *** **************** ****************

tr-1 TGTGAGTGTAAGAAAAGAGAAAACCCGGAGGAGAGATACAGCGGAAGGTACTGCGAGTGC 1602

tr-2 TGTGAATGTAAGAAAAGAGAAAACCCGGAGGAGAGATACAGCGGAAGGTACTGCGAGTGC 1602

tr-3 TGTGAATGTAAGAAAAGAGAAAACCCGGAGGAGAGATACAGCGGAAGGTACTGCGAGTGC 1620

ESTa TGTGAGTGTAAGAAAAGAGAAAACCTGGAGGAGAGATACAGCGGAAATTACTGCGAGTGC 1620

ESTb TGTGAGTGTAAGAAAAGAGAAAACCCGGAGGAGAGATACAGCGGAAGGTACTGCGAGTGC 1602

***** ******************* ******************** ************

tr-1 GACAACTTCAGCTGCGACCGCTTTAACAACAAGCTCTGTGGAGGTCACGGTCGATGTATG 1662

tr-2 AACAACTTCAGCTGCGACCGCTTTAACAACAAGCTCTGTGGAGGTCACGGTCGATGTGTG 1662

tr-3 AACAACTTCAGCTGCGACCGCTTCAACAACAAGCTCTGTGGAGGTCATGGTCGATGTATG 1680

ESTa GACAACTTGAGCTGCGACCGCTTTAACAACAATATCTGTGGAGGTCACGGTCGATGTGTG 1680

ESTb AACAACTTCAGCTGCGACCGCTTCAACAACAAGCTCTGTGGAGGTCACGGTCGATGTATG 1662

******* ************** ******** ************* ********* **

tr-1 TGCGGGCAGTGTGCATGTGAATTTAACTACGCCGGCAGTGCATGCCAATGCTCTATGGAT 1722

tr-2 TGCGGGCAGTGTGCATGTGAATTTAACTATGCCGGCAGTGCATGCCAATGCTCTATGGAT 1722

tr-3 TGCGGGCAGTGTGCATGTGAATTTAACTACGCCGGCAGTGCATGCCAATGCTCTATGGAT 1740

ESTa TGCGGGCAGTGTAAATGTGAATTTAACTATGCCGGCAATGCATGCCAATGCTCTATGGAT 1740

ESTb TGCGGGCAGTGTGCATGTGAATTTAACTACGCCGGCAGTGCATGCCAATGCTCTATGGAT 1722

************ *************** ******* **********************

tr-1 ACCTCGAGCTGTCTGGCCAGTAACAAACTGATCTGTAATGGACACGGCATCTGTGAATGT 1782

tr-2 ACCTCGAGCTGTCTGGCCAGTAACAAACTGATCTGTAATGGACACGGCATCTGTGAATGT 1782

tr-3 ACCTCGAGCTGTCTGGCCAGTAACAAACTGATCTGTAATGGACACGGCATCTGTGAATGT 1800

ESTa ACCTCGAGCTGTCTGGCCAGTAACAAACTGATCTGTAATGGACACGGCATCTGTGAATGT 1800

ESTb ACCTCGAGCTGTCTGGCCAGTAACAAACTGATCTGTAATGGACACGGCATCTGTGAATGT 1782

************************************************************

tr-1 GGACAGTGTAAATGTTTGGACAATTGTCAAGGTCCAACCTGTGAAATCTGCCTTACC**TGA** 1842

tr-2 GGACAGTGTAAATGTTTGGACAATTGTCAAGGTCCAACCTGTGAAATCTGCCTAACC**TGA** 1842

tr-3 GGACAGTGTAAATGTTTGGACAATTGTCAAGGTCCAACCTGTGAAATCTGCCTAACC**TGA** 1860

ESTa GGACAGTGTAAATGTTTGGACAATTGTCAAGGTCCAACCTGTGAAATCTGCCTTACC**TGA** 1860

ESTb GGACAGTGTAAATGTTTGGACAATTGTCAAGGTCCAACCTGTGAAATCTGCCTAACC**TGA** 1842

***************************************************** ******

tr-1 AACAGTGGTGTGAAAAGGAAAAAACTGACACCTGAAAGCATATCCCTCAATTTATACTGT 1902

tr-2 AACAGTGGTGTGAAAAGGAAAAAACTGACACCTGAAAGCATATCCCTCAATTTATACTGT 1902

tr-3 AACAGTGGTGTGAAAAGGAAAAAACTGACACCTGAAAGCATATCCCTCAATTTATACTGT 1920

ESTa AACAGTGGTGTGAAAAGGAAAAAACTGACACCTGAAAGCATATCCCTCAATTTATACTGT 1920

ESTb AACAGTGGTGTGAAAAGGAAAAAACTGACACCTGAAAGCATATCCCTCAATTTATACTGT 1902

************************************************************

tr-1 ACATCAT-----GACTCATGTTATACAT-------------------------------- 1925

tr-2 ACATCATATTGAGACACATGTTATACA-------------------------------- 1929

tr-3 ACATCATATTGAGACACATGTTATACA--------------------------------- 1947

ESTa ACATCAT-----GACTCATGTTATACATTTTAATTAATGAGTGTCATCATAGGTCAGTAA 1975

ESTb ACATCATATTGAGACACATGTTATACATTTTAATTATTGAGTGTCATCATAGGTCAGTAA 1962

******* *** ***********

tr-1 ------------------------------------------------------------

tr-2 ------------------------------------------------------------

tr-3 ------------------------------------------------------------

ESTa GATGATGTCGGTTGCTGCGATATGTTTGCATGTTATTTTAACCTACAAAAAAGGTTTTGA 2035

ESTb GATGATGTTGGTTGATGCAATATGTTTGCATGTTATTATAACCTACGAAACAGGTTATGA 2022

tr-1 ------------------------------------------------------------

tr-2 ------------------------------------------------------------

tr-3 ------------------------------------------------------------

ESTa TCTTACAGTATATACAAGATTTATACAATTTATATTTTTAGAAATGCTGTGATGCAAAAA 2095

ESTb TCTTACAGTATATACAAGATTTATACAATTTATATTTTTAGAAATGCTGTGATGCAAAAA 2082

tr-1 ------------------------------------------------------------

tr-2 ------------------------------------------------------------

tr-3 ------------------------------------------------------------

ESTa TACTACACAGAGTGAATTGTCTTAATGCCTGGGATTAAATATGGGTGTTTCAAAAAAAAA 2155

ESTb TACTACACAGAGTGAATTGTCTTAATGCCTGGGATTAAATATGGGTGTTTCAAAAAAAAA 2142
