# Supplementary material for: Identification of multiple integrin β1 homologs in zebrafish (Danio rerio)
Source: BMC Cell Biol. 2006 Jun 20;7:24. doi: 10.1186/1471-2121-7-24 (PMC1538996; doi:10.1186/1471-2121-7-24)
Supplement: Additional File 2 — Protein sequence alignment of zebrafish β1 sequences with human and Ciona β1 orthologs. Alignment was performed using Clustal X and is displayed using Boxshader. Levels of sequence conservation are indicated (>50% identical, red; conservative substitutions, blue). Note that the refined Ciona β1 sequence [8] is missing the signal peptide, the PSI domain and part of the hybrid domain. [file 1471-2121-7-24-S2.doc]

Additional File 2

Hs_b1 1 MNLQPIFWIGLISSVCCVFAQTDENRCLKANAKSCGECIQAGPNCGWCTNSTFLQEGMPT
Dr_b1-1 1 MDLKLLFISALLGIISCSRAQQEGNECIKANALSCGECIQVGDKCGWCTDAEFLKQGEPT
Dr_b1-2 1 MDVRLLLISVLLG---LSRAQQDGNECTKASAQSCGECIQAGEKCGWCTDEGFLKQGEQK
Dr_b1tr-1 1 MDITVLLLSALLGFVSDVGANTDSNPCISANAKTCGECIQIGPQCVWCKDPDFK------
Dr_b1tr-2 1 MDITVLLLSALLGFVSDVGANTDSNPCISANAKTCGECIQIGPQCVWCKDPDFK------
Dr_ESTb 1 MDITVLLLSALLGFVSYVGANTDSNPCISANAKTCGECIQIGPQCVWCKDPDFK------
Dr_ESTa 1 MDITVLLLSALLGFVSDVGANTDSNPCISANAKTCGECIQIGPQCVWCKDPDFK------
Dr_b1tr-3 1 MDITVLLLSALLGFVSDVGANTDSNPCISANAKTCGECIQIGPQCVWCKDPDFK------
Dr_b1-3 1 MKMKLLLLSALLGFVFHVGAKTDSNPCISANAKTCGECIQIGPQCVWCKDPDFK------
Ci_b1 1 ------------------------------------------------------------


Hs_b1 61 SARCDDLEALKKKGCPPDDIENPRGSKDIKKNKNVTNRSKGTAEKLKPEDIHQIQPQQLV
Dr_b1-1 61 SARCDELESLKKRGCAEAKIENPHGSQRILKNTPVTNRKKG-AEKLRPEDITQIQPQKLS
Dr_b1-2 58 STRCDEIEALEKKGCSKASIENPRGKITIVKNQPVTNRTKN-GAKLKPDQITQIQPQQLS
Dr_b1tr-1 55 PSRCDDIESMAKAGCTADGVENPRGAVTIDKNKPVTNRKTDGGQNLRPDEITQIQPQKVT
Dr_b1tr-2 55 PSRCDDIESMAKAGCTADGVENPRGAVTIDKNKPVTNCKIDGGQNLRPDEITQIQPQKVT
Dr_ESTb 55 PSRCDDIESMAKAGCTADGVENPRGAVTIDKNKPVTNRKTDGGQNLRPDEITQIQPQKVK
Dr_ESTa 55 PSRCDDIESMAKAGCTADGVENPRGAVTIDKNKPVTNRKTDGGQNLRPDEITQIQPQKVK
Dr_b1tr-3 55 PSRCDDIESMAEAGCTADGVENPRGAVTIDKNKPVTNRKTDGGQNLRPDEITQIQPQKVT
Dr_b1-3 55 PSRCDDIESMAKAGCTADGVENPRGAVTIDKNKPVTNRKTDGGQNLRPDEITQIQPQKVT
Ci_b1 1 ---------------------SNVGESAVGK-------------------ATQIRPQIIN


Hs_b1 121 LRLRSGEPQTFTLKFKRAEDYPIDLYYLMDLSYSMKDDLENVKSLGTDLMNEMRRITSDF
Dr_b1-1 120 LQLRSGEPQNIKLKFKRAEDYPIDLYYLMDLSYSMKDDLENVKNLGTSLMKEMSKITSDF
Dr_b1-2 117 LNLRSGEAQKFTLKFKRAEDYPIDLYYLMDLSYSMKDDLENVKNLGTDLMKEMQKITSDF
Dr_b1tr-1 115 LNLRSGEAQKFTLKFKRAEDYPIDLYFLMDLSHSMLSNLENLKNLGFELAKEMKDITKDL
Dr_b1tr-2 115 LNLRSGEAQKFTLKFKRAEDYPIDLYFLMDLSHSMLSNLENFKNLGTELANEMKDITKDL
Dr_ESTb 115 LNLRSGEAQKFTLKFKRAEDYPIDLYFLMDLSHSMLSNLENFKNLGTELANEMKDITKDL
Dr_ESTa 115 LNLRSGEAQKFTLKFKRAEDYPIDLYFLMDLSHSMLSNLENFKNLGTELANEMKDITKDL
Dr_b1tr-3 115 LNLRSGEAQKFTLKFKRAEDYPIDLYFLMDLSHSMLSNLENLKNLGTELANEMKDITKDL
Dr_b1-3 115 LNLRSGEAQKFTLKFKRAEDYPIDLYFLMDLSHSMVSNLENVKKLGTELANEMKDITKDL
Ci_b1 21 SRIRPGDPLNIELSFKQAEDYPVDLYYLLDLSKSMENDLNSLRALGRELGTSMQNITRDF


Hs_b1 181 RIGFGSFVEKTVMPYISTTPAKLRNPCTSEQNCTTPFSYKNVLSLTNKGEVFNELVGKQR
Dr_b1-1 180 RIGFGSFVEKTVMPYISTTPAKLLNPCTGDQNCTSPFSYKNVLKLTSNGQRFNSLVGQQQ
Dr_b1-2 177 RIGFGSFVEKTVMPYISTTPAKLLNPCTSDQNCTSPFSYKNVLSLTDDGSQFNSLVSRQQ
Dr_b1tr-1 175 RIGFGSFFRK---------PSIQTNPCFPD-NCIAPFSYFNVLSLTDDHALFTQEISKLK
Dr_b1tr-2 175 RIGFGSFFRK---------PSIQTNPCFPD-NCIAPFSYFNVLSLTDDHALFTQEISKLK
Dr_ESTb 175 RIGFGSFFRK---------PSIQTNPCFPD-NCIAPFSYFNVLSLTDDHALFTQEISKLK
Dr_ESTa 175 RIGFGSFFRK---------PSIQTNPCFPD-NCIAPFSYFNVLSLTDDHALFTQEISKLK
Dr_b1tr-3 175 RIGFGSFFRK---------PSIQTNPCFPD-NCIAPFSYFNVLSLTDDHALFTQEISKLK
Dr_b1-3 175 HIGFGSFLEKLVMPYILMTPKYLKNPCFPS-DCTAPFSYKNVLSLTDNHGLFTQEVSKQK
Ci_b1 81 RLGFGSFIDKTVMPYISTVPAKIRNPCNDKAPCVPTYSFHNDLPLTPEIDAFVNSVNNVT


Hs_b1 241 ISGNLDSPEGGFDAIMQVAVCGSLIGWR-NVTRLLVFSTDAGFHFAGDGKLGGIVLPNDG
Dr_b1-1 240 ISGNLDSPEGGFDAIMQVAVCGEHIGWR-NVTRLLVFSTDAGFHFAGDGKLGGIVLPNDG
Dr_b1-2 237 ISGNLDSPEGGFDAIMQVAVCGNQIGWR-NVTRLLVFSTDAGFHFAGDGKLGGIVLPNDG
Dr_b1tr-1 225 TSGNLDSSEAGLEALMQAAVCTDVIGWR-NATRVLVFFTDAGLRFSGDGKRGGIVRLNDG
Dr_b1tr-2 225 TSGNLDSSEAGLEALMHAAVCTDVIGWR-NVTRVLVFFTDAGLRFSGDGKRGGIVRLNDG
Dr_ESTb 225 TSGNLDSSEAGLEALMQAAVCTDVIGWR-NVTRLLVFFTDAGLRFSGDGKRGGIVRLNDG
Dr_ESTa 225 TSGNLDSSEAGLEALMQAAVCTDVIGWR-NATRVLVFFTDAGLRFSGDGKRGGIVHLNDG
Dr_b1tr-3 225 TSGNLDSSEAGLEALMQAAVCTDVIGWR-NVTRVLVFITDAGLRFSRDGKRGDIVRLNDG
Dr_b1-3 234 TSGNLDAPEAGFDAIMQAAVCTDVIGWR-NVTRLLVFSTDAGFHLAGDGKLGGIVRPNDG
Ci_b1 141 HSSNLDNPEGGLDAMMQAIVCKEKINWRKDATHLLVYSTDASFHYAGDGKLGGIVLPNDG


Hs_b1 300 QCHLENN-MYTMSHYYDYPSIAHLVQKLSENNIQTIFAVTEEFQPVYKELKNLIPKSAVG
Dr_b1-1 299 RCHLEND-MYTMSHYYDYPSIAHLVQKLSENNIQTIFAVTEEFQPVYKELKNLIPKSAVG
Dr_b1-2 296 KCHLQDN-IYTMSHYYDYPSIAHLVQKLSENNIQTIFAVTEEFQPVYQELKNLIPKSAVG
Dr_b1tr-1 284 KCLLDDN-MYTRSDYSDYPSLSQLVDTVTDNSIHTIFAVTEQFQDLYQELSAKVPNSAVG
Dr_b1tr-2 284 KCLLEDN-MYTRSDYSDYPSLSQLVDTVTDNSIHTIFAVTEQFRDLYQELSAKVPNSAVG
Dr_ESTb 284 KCLLDDN-MYTRSDYSDYPSLSQLVDTVTDNSIHTIFAVTEQYRDLYQELSAKVPNSAVG
Dr_ESTa 284 KCLLDDN-MYTRSDYSDYPSLSQLVDTVTDNSIYTIFAVTEQFRDLYQELSAKVPNSAVG
Dr_b1tr-3 284 KCLLDDN-MYTRSDYSDYPSLSQLVDTVTDNSIHTIFAVTEQFQDLYQELSAKVPNSAVG
Dr_b1-3 293 KCHLDNN-MYTMSNYFDYPTISQLVDTLSGNNIQTIFAVTEEIREIYQELSALIPKSAVG
Ci_b1 201 NCYLDDNGHYYNANAMDYPSIGHLVRKITSHNIQPIFAVTTSVIQTYTNLQKMIPKSVVG

Hs_b1 359 TLSANSSNVIQLIIDAYNSLSSEVILENGKLSEGVTISYKSYCKNGVNGTGENGRKCSNI
Dr_b1-1 358 TLSANSSNVINLIVDAYNSLSSEVILENSKLPEGVTITYQSRCKNGVVNEGESGRKCSNI
Dr_b1-2 355 TLTSDSNNVIKLIIDAYNSLSSEVILENSKLPEGVSISYVSHCKNGVSGTGDTGRKCSNI
Dr_b1tr-1 343 TFSTSGDNLAKLVIDALIPLSSEVIVENSKLPDGVSISYVSHCKNGVNGRGEDGRKCSNI
Dr_b1tr-2 343 TFSTSGDNLAKLVIDALIPLSSEVIVENSKLPDGVSISYVSHCKNGVNGRGEDGRKCSNI
Dr_ESTb 343 TLSTSGDNLAKLVIDALIPLSSEVIVENSKLPDGVSISYVSHCKNGVNGRGEDGRKCSNI
Dr_ESTa 343 TLSTSGDNLAKLVIDALIPLSSEVIVENSKLPDGVSISYVSHCKNGVNGRGEDGRKCSNI
Dr_b1tr-3 343 TLSTSGDNLAKLVIDALISLSSKVIVENSKLPDGVSISYVSHCKNGVNGRGEDGRKCSSI
Dr_b1-3 352 ILSTSSSNVIKLIIDAYNSLSSEVILENSKLPDGVSISYVSHCKNGVSGTGDNGRKCSNI
Ci_b1 261 ELSGDSSNIIQLIQNAYNDLKGQVLLEIRAPPEVTVSSQTAHCQN----QTIPGMQCEGV


Hs_b1 419 SIGDEVQFEISITSNKCPKK-DSDSFKIRPLGFTEEVEVILQYICECECQSEGIPESPKC
Dr_b1-1 418 SIGDEVSFNINITAQGCPKQGKTETIKIKPLGFTEEVEITLSFICECECHKHAMKNSPLC
Dr_b1-2 415 SIGDEVAFEVAITAKGCPLNGKSETMKIKLLGFTEEVEVVLNFICECECHKDGIKNSPVC
Dr_b1tr-1 403 SIGDEVLFDIEITAKGCPSKGKPETIKIKPLGFSEEVEILLNYICECECHKGGIKNSPEC
Dr_b1tr-2 403 SIGDEVLFDIEITAKGCPSKGKPETIKIKPLGFSEEVEILLNYICECECHKDGIKNSPKC
Dr_ESTb 403 SIGDEVLFDIEITAKGCPSKGKPETIKIKPLGFSEEVEIFLNYICECECHKDGIKNSPEC
Dr_ESTa 403 SIGDEVLFDIEITAKGCPSKGKPETIKIKPLGLSEEVEIFLNYICECECHKDGIKNSPKC
Dr_b1tr-3 403 SIGDEVLFDIEITAKGCPSKGKPETIKIKLLGFSEEVEILLNYICECECHKDGIKNSPEC
Dr_b1-3 412 SIGDEVLFDIEITAKGCPSKGKPETIKIKPLGFSEEVEILLNYICECECHKDGIKNSPKC
Ci_b1 317 KLGDIVNFTFTLSTEKCLKS--PVQVLVSPYGYNEVVTINVESHCDCQCEETQAPTTN-C


Hs_b1 478 HEGNGTFECGACRCNEGRVGRHCECSTDEVN-SEDMDAYCRKENSSEICSNNGECVCGQC
Dr_b1-1 478 HNGNGSFECGACRCNKGRVGRQCECRKDEVS-TEDLDKNCRKDNGTDICSNNGECVCGTC
Dr_b1-2 475 HFGNGTLECGACRCNEGRIGRVCECSKDEVR-TEDLDANCRMDNGTDICSNNGDCVCGTC
Dr_b1tr-1 463 SGGQGTLECGVCRCNEGRSGRICECTQD-------LDAYCQMDMSSGICSNNGECVCGTC
Dr_b1tr-2 463 SGGQGTLECGVCRCNEGRSGRICECTQD-------LDAYCQMDMSSGICSNNGECVCGTC
Dr_ESTb 463 SGGQGTLECGVCRCNEGRSGRICECTQD-------LDAYCQMDMSSGICSNNGECVCGTC
Dr_ESTa 463 SGGQGTLECGVCRCNEGRSGRLCECSHDKLL-ADDLDAYCRMNNGTEVCSNNGECVCGIC
Dr_b1tr-3 463 SGGQGTLECGVCRCNEGRLGRLCECSHDEVL-ADDLDAYCRMNNGTEVCSNNGECVCGTC
Dr_b1-3 472 SGGQGTLECGVCRCNEGRLGRLCECSHDEVL-ADDLDAYCRMNNGTEVCSNNGECVCGTC
Ci_b1 374 S-GHGVYECGSCVCETGFTGLDCSCDQKDVLGIESYLANCTDPATGVVCNSGGECQCGSC


Hs_b1 537 VCRKRDNTNEIYSGKFCECDNFNCDRSNGLICGGNGVCKCRVCECNPNYTGSACDCSLDT
Dr_b1-1 537 ECKKRENPEERYSGKYCECDNFNCDRSNNKLCGGHGRCECRVCVCDANYTGSACDCSLDT
Dr_b1-2 534 ECKKRDNPEERYSGKFCECDNFNCDRSNNKLCGGHGRCDCRKCICDANYTGSACDCSLDT
Dr_b1tr-1 516 ECKKRENPEERYSGRYCECDNFSCDRFNNKLCGGHGRCMCGQCACEFNYAGSACQCSMDT
Dr_b1tr-2 516 ECKKRENPEERYSGRYCECNNFSCDRFNNKLCGGHGRCVCGQCACEFNYAGSACQCSMDT
Dr_ESTb 516 ECKKRENPEERYSGRYCECNNFSCDRFNNKLCGGHGRCMCGQCACEFNYAGSACQCSMDT
Dr_ESTa 522 ECKKRENLEERYSGNYCECDNLSCDRFNNNICGGHGRCVCGQCKCEFNYAGNACQCSMDT
Dr_b1tr-3 522 ECKKRENPEERYSGRYCECNNFSCDRFNNKLCGGHGRCMCGQCACEFNYAGSACQCSMDT
Dr_b1-3 531 ECKKRDNPEERYSGKFCECDNFSCDRSNNKLCGGHGRCECKKCICDANYTGSACDCPLDT
Ci_b1 433 ICKQYAN--KKIDGKYCECDNTTCDRAGARVCNGFGKCNCGVCECQNGWKGKACDCTLDQ


Hs_b1 597 STCEASN------GQICNGRGICECGVCKCTDP---KFQGQTCEMCQTCLGVCAEHKECV
Dr_b1-1 597 STCLASN------KQICNGRGICECGTCRCTDP---KFQGPTCEICPTCPGVCTEHKECV
Dr_b1-2 594 STCLASN------KQICNGRGNCECGACKCTDT---KFQGPTCEICPTCPGVCTEHKDCV
Dr_b1tr-1 576 SSCLASN------KLICNGHGICECGQCKCLD----NCQGPTCEICLT------------
Dr_b1tr-2 576 SSCLASN------KLICNGHGICECGQCKCLD----NCQGPTCEICLT------------
Dr_ESTb 576 SSCLASN------KLICNGHGICECGQCKCLD----NCQGPTCEICLT------------
Dr_ESTa 582 SSCLASN------KLICNGHGICECGQCKCLD----NCQGPTCEICLT------------
Dr_b1tr-3 582 SSCLASN------KLICNGHGICECGQCKCLD----NCQGPTCEICLT------------
Dr_b1-3 591 ATCLAST------NEICNGRGKCECGVCKCDE----NYEGPTCEICPTCPRICTERKDCV
Ci_b1 491 TECYDLSPDAVDSSKPCNGNGECECGQCVCNSRGGAKFRGQYCKTKPLV--ICDIHKDCI


Hs_b1 648 QCRAFNKGEK-KDTCTQECSYFNITKVESRDKLPQPVQPDPVSHCKEKDVDDCWFYFTYS
Dr_b1-1 648 QCRAFGTGEK-KDTCKRDCSYFNLIEVEDRDKLPQPVQAFPLMHCKERDARDCWFYYTYA
Dr_b1-2 645 QCRAFGTGDK-KDTCEEQCSYFTMKVVKKKEDLPQPNDQPIINHCKERDANDCWFFFTYA
Dr_b1tr-1 ------------------------------------------------------------
Dr_b1tr-2 ------------------------------------------------------------
Dr_ESTb ------------------------------------------------------------
Dr_ESTa ------------------------------------------------------------
Dr_b1tr-3 ------------------------------------------------------------
Dr_b1-3 641 ECRHFGTGSK-QKTCEEDCKSYSIKKVKTKEDLPPPNIN----HCKERDVDDCWIFFTSS
Ci_b1 549 QCKAWKTGNYNTTECEKQCTKYNVTKMSKEYQYSG-----YTNECRFTDLTDDCNYQATF

Hs_b1 707 VNGNNEVMVHVVENPECPTGPDIIPIVAGVVAGIVLIGLALLLIWKLLMIIHDRREFAKF
Dr_b1-1 707 VNNNTEKEVHVVKTMECPPGPDIIPIVAGVVAGIVLIGLALLLIWKLLMIIHDRREFAKF
Dr_b1-2 704 TRNDSSVMVHVAEELECPSGPDIIPIVAGVVAGIVLIGLALLLIWKLLMIIHDRREFAKF
Dr_b1tr-1 ------------------------------------------------------------
Dr_b1tr-2 ------------------------------------------------------------
Dr_ESTb ------------------------------------------------------------
Dr_ESTa ------------------------------------------------------------
Dr_b1tr-3 ------------------------------------------------------------
Dr_b1-3 696 IEKDGSIQVYVAENRECPSGPDIIPIVAGVVAGIVLIGLALLLIWKLLMVIHDRREFDKF
Ci_b1 604 EEIDGIIMVEVEPDKTCTTYANPVYIIIGIIAAIVGIGLAILLIWKLLTSIKDAREYKNF


Hs_b1 767 EKEKMNAKWDTGENPIYKSAVTTVVNPKYEGK-----
Dr_b1-1 767 EKEKMNAKWDTGENPIYKSAVTTVINPKYEGK-----
Dr_b1-2 764 EKEKMNAKWDAGENPIYKSAVTTVVNPKYEGK-----
Dr_b1tr-1 -------------------------------------
Dr_b1tr-2 -------------------------------------
Dr_ESTb -------------------------------------
Dr_ESTa -------------------------------------
Dr_b1tr-3 -------------------------------------
Dr_b1-3 756 EKEKNNAKWDTGENPIYKSAVTTVVNPRYEGK-----
Ci_b1 664 QKESQNPKWQGGENPIFKKATSTFKNPMYSGGKTAGN
